# Organic formulation modulating crop health, yield and managing anthracnose-twister and Stemphylium blight of onion

**DOI:** 10.3389/fpls.2026.1774060

**Published:** 2026-05-11

**Authors:** Ram Dutta, Jayalakshmi K, Satish Kumar, Auji Radhakrishna, Thangasamy Arunachalam, Pranjali V. Bhadane, Vijay Mahajan

**Affiliations:** Indian Council of Agricultural Research (ICAR)-Directorate of Onion and Garlic Research, Pune, Maharashtra, India

**Keywords:** *Allium cepa*, anthracnose-twister, bio-stimulants, LC-MS, microbial diversity, non-VOCs, organic formulation, Stemphylium blight

## Abstract

In India, onion productivity during the *Kharif* season (June-October) is considerably lower than in the *Rabi* season (December-April), largely due to the high incidence of anthracnose-twister and Stemphylium blight. To promote sustainable disease management, four organic formulations (DOGROF-1 to DOGROF-4) were developed using locally available natural bio-resources and evaluated under field conditions for three years (2021-2023) at ICAR-Directorate of Onion and Garlic Research, Pune, using the popular onion varieties Bhima Super (*Kharif*) and Bhima Shakti (*Rabi*). Foliar application of DOGROF-3 significantly improved plant height, pseudostem girth, and leaf number, resulting in yield increases of 34.23% in *Kharif* and 29.17% in *Rabi*. It also reduced anthracnose-twister and Stemphylium blight severity by 49.62% and 48.09%, respectively. DOGROF-3 also exhibited a higher incremental cost-benefit ratio (ICBR) with values of 1:33.65 (*Kharif*) and 1:34.04 (*Rabi*), highlighting its economic feasibility. Microbial profiling revealed DOGROF-3 harbored the highest populations of beneficial bacteria (3×10^8^ cfu/mL), fungi (4 × 10^5^ cfu/mL) and actinomycetes (8 × 10^5^ cfu/mL). LC-MS analysis led to the putative identification of 30 bioactive compounds, including hexylglutathione conjugates, indoleacrylic acid, gibberellins, caffeyl alcohol, isorhamnetin-3-O-rhamnoside, and candicidin D. Literature reports indicate that several of these metabolites are associated with plant growth promotion and antifungal activity, suggesting their possible role in disease suppression and improved plant performance. The nutrient profiling of bioformulations indicated that, although DOGROF-3 contained comparatively lower macronutrients (N- 0.09%, P- 0.97%, K-3.42%), it was markedly enriched in essential micronutrients, particularly zinc (20.25 ppm), manganese (8.62 ppm) and copper (7.18 ppm), which are known to be associated with enhanced enzyme activity, photosynthetic performance, and nutrient-use efficiency. These findings suggest DOGROF-3 offers an eco-friendly, and economically viable alternative to chemical fungicides, supporting sustainable crop health and disease management in onion production.

## 1 Introduction

Onion (*Allium cepa* L.) is one of the most important vegetable crops cultivated in India, valued for its distinctive flavor, pungency, and health-promoting properties. India has emerged as the leading global producer of onion, recording a production of 31.7 million tonnes (Mt) in 2022 and 30.2 Mt in 2023, surpassing China, which produced 24.5 Mt and 24.9 Mt during the respective years (FAO, 2025). However, this increase in production has largely been achieved through the intensive use of agrochemicals, since onion is a high input–intensive crop (Bhardwaj et al., 2025). Modern onion production systems rely heavily on synthetic fertilizers and chemical pesticides to maintain crop health and attain high yields (Li et al., 2025). Studies have shown that farmers commonly apply substantial quantities of nutrients to sustain rapid vegetative growth and bulb development in onion crop. For instance, drip-irrigated onion fields have been reported to receive an average of approximately 649.57 kg of fertilizer nutrients per hectare, reflecting the crop’s heavy dependence on external nutrient inputs (Kale et al, 2024). Additionally, onions are highly susceptible to destructive fungal diseases such as anthracnose-twister (caused by *Colletotrichum* spp. and/or *Fusarium* spp.), and Stemphylium blight (*Stemphylium vesicarium*), which often necessitate repeated applications of chemical fungicides for effective disease management. The fungal pathogens of onion often infect both foliage and bulbs, leading to severe yield losses by 50–100% under favorable conditions (Dutta et al., 2022). Stemphylium leaf blight, caused by *S. vesicarium*, has been reported to reduce onion yield and quality by as much as ~90% in susceptible cultivars under severe infection (Chandel et al., 2022).

The onion growing farmers in Maharashtra, a major onion-growing state of India, heavily relies on fungicide spray and apply nearly seven crop protection sprays per crop cycle to keep pathogen pressure under control (Kale et al., 2024). Although chemical fertilizers and fungicides application increase immediate yields, their indiscriminate application in cropping systems contributes to soil health deterioration, nutrient imbalance, and reduced crop productivity (Khandare et al., 2020). This also increases production costs, accelerates the development of fungicide-resistant strains, and poses risks to human health and the environment. Furthermore, the prolonged application of chemical inputs to onion fields has contributed to the emergence of pathogen resistance and reduced input-use efficiency in onion production system (Jwaideh et al., 2022; Qiu et al, 2025). Fungicide resistance in onion pathogens has been increasingly reported with *Stemphylium vesicarium* in New York exhibiting mutations that confer reduced sensitivity to multiple fungicide groups, including QoI fungicides such as azoxystrobin and pyraclostrobin (Hay et al., 2019).

Owing to the well-documented deleterious effects of synthetic agrochemicals on soil health and their potential residue-related risks to human food consumption, the use of microbial inoculants and organic bioformulations has emerged globally as promising alternative. Such formulations can concurrently promote crop growth, enhance tolerance to biotic and abiotic stresses, and suppress disease incidence through synergistic processes including microbial antagonism, induced systemic resistance, and improved soil and plant nutrient dynamics (Anandham et al., 2015). A recent field study in onion demonstrated that the combined application of organic fertilizers (vermicompost and rabbit manure) with the biofertilizer *Azotobacter chroococcum* significantly enhanced plant growth, leaf number, bulb diameter and weight, and total yield (up to ~65 t h^-1^) compared with non-inoculated controls (Ali et al., 2025). This approach also improved soil properties, including organic matter content, pH, and electrical conductivity highlighting the potential of microbial bioformulations as a sustainable alternative to synthetic fertilizers in onion production systems. Studies on application of *Trichoderma* strains like *T. longibrachiatum* and *T. asperellum* consistently increased bulb yield by up to 34%, likely through increased chlorophyll content and IAA production and improved nutrient uptake, compared with untreated control plants (Dutta et al., 2024). Moreover, the combined use of arbuscular mycorrhizal fungi with *T. harzianum* reduced disease severity and promoted onion growth under pathogen pressure, indicating synergistic microbial effects on plant health (Yagmur et al, 2024).

While microbial inoculants have demonstrated clear benefits in promoting plant growth and suppressing pathogens, their field performance is often inconsistent due to poor survival, limited establishment, and strong dependence on soil type, climate, and native microbial competition. The introduced microbial strains frequently fail to persist or function optimally under open-field conditions, particularly in intensively managed cropping systems. In contrast, organic bioformulations offer a more robust and resilient approach by combining fermentable organic substrates, plant-derived bioactive compounds, and naturally enriched native microbial consortia within a single input. Such formulations provide readily available carbon and nutrient sources that support microbial proliferation and activity, thereby enhancing the survival and functional expression of beneficial microorganisms. Additionally, botanicals rich in secondary metabolites contribute direct antimicrobial effects while simultaneously acting as elicitors of plant defense responses. Importantly, organic formulations tend to promote native microbial communities rather than relying solely on the introduction of external strains, leading to improved rhizosphere stability and long-term soil health. The multifunctional nature of organic bioformulations enables them to simultaneously improve nutrient availability, stimulate plant growth through phytohormone-like compounds, enhance induced systemic resistance, and suppress a broad spectrum of pathogens. Consequently, organic bioformulations are often better suited for sustainable, low-input, and organic production systems where consistent performance and ecological compatibility are critical. This highlights the need for developing integrated organic bioformulations that combine fermentable organic substrates, plant-derived bioactive compounds, and naturally enriched microbial consortia into a single, scalable input capable of simultaneously enhancing crop growth and suppressing diseases under field conditions.

Hence, there is growing interest in complex organic bioformulations that go beyond single-input amendments or individual microbial inoculants. Traditional and farmer-developed formulations prepared through natural fermentation of plant biomass, organic substrates, and animal-derived inputs represent an integrated strategy that simultaneously supplies nutrients, introduces diverse native microbial consortia, and delivers plant-derived bioactive compounds with antimicrobial and elicitor properties (Yuvasri et al, 2024). Such formulations combine fermentable carbon sources (e.g., cereal flours and jaggery), botanicals rich in secondary metabolites (e.g., neem, *Calotropis*, karanj, garlic, chilli), and biologically active amendments (cow dung, cow urine, virgin soil, lime), creating a multifunctional input capable of enhancing plant vigor while suppressing pathogens. Each component of such organic formulations plays a discrete and complementary role in improving soil health and plant performance. Gram flour serves as a readily available carbon and protein source, stimulating microbial proliferation and accelerating nutrient mineralization. *Calotropis* leaves are rich in bioactive secondary metabolites, including calotropin and uscharin, which exhibit antifungal, insecticidal, and antimicrobial properties (Bhardwaj et al., 2025). Neem leaves contribute well-documented bioactive compounds such as azadirachtin and nimbin that impart pesticidal, nematicidal, and antimicrobial effects, thereby aiding in disease and pest suppression (Dhakad et al, 2025). Cow dung acts as a natural reservoir of macro- and micronutrients as well as beneficial microbial communities that enhance soil fertility and biological activity (Ram, 2023). Cow urine acts as a nutrient-rich organic input by supplying plant-available nitrogen and essential macro- and micronutrients, along with humic and fulvic acid-type compounds that can stimulate growth and nutrient uptake (Sodani and Kumar, 2017; Yemata and Mengistu, 2024). Jaggery functions as an easily assimilable carbon source that enhances microbial metabolism, supports fermentation efficiency, and promotes the establishment of beneficial microbial consortia (Palekar, 2006; Sharma et al., 2021; Kumar et al., 2025). The synergistic interaction among these constituents enhances soil fertility, microbial diversity, nutrient uptake, and plant vigor while suppressing disease incidence, thereby improving onion yield and bulb quality under organic cultivation.

Evaluating the traditional organic formulations for their effects on crop health and disease management in onion is essential for bridging indigenous knowledge with modern scientific validation. Although farmer adoption of organic based inputs is increasing, systematic scientific studies remain limited, especially regarding their microbial composition, nutrient profile, bioactive non-volatile compounds, and field-level efficacy against major onion diseases. Despite the widespread field application of organic bioformulations, detailed information on their biochemical composition and active constituents remains limited. Most studies evaluate such formulations primarily through agronomic responses, with little emphasis on resolving their underlying metabolite profiles. In the present study, we employed high-resolution LC-MS-based metabolomic profiling to comprehensively characterize the biochemical constituents of four distinct bioformulations used under field conditions. Notably, the formulations used in this study differed in their processing as only the three bioformulation were fermented whereas one bioformulations was used as non-fermented, allowing us to systematically assess the influence of fermentation on biochemical composition. This approach enabled the identification of both shared and formulation-specific metabolites, thereby revealing common biochemical signatures as well as unique compounds potentially generated or enriched through fermentation. In this context, the present study applies untargeted LC-MS profiling to comparatively evaluate fermented and non-fermented organic bioformulations, offering new insights into their biochemical diversity and providing a molecular basis for understanding their potential functional roles in agricultural systems. Therefore, the present study was undertaken to: (i) evaluate the effect of organic based formulations on yield and disease incidence (anthracnose-twister and Stemphylium blight) of onion under field conditions (ii) characterize the microbial and nutrient composition of the formulations; and (iii) analyze the bioactive non-volatile compounds through LC-MS to establish possible links with disease suppression and plant growth promotion.

## 2 Materials and methods

### 2.1 Preparation of organic formulation

DOGROF-1: The formulation was prepared by mixing gram flour (1 kg), crushed *Calotropis* leaves (1 kg), neem leaves (1 kg), cow dung (1 kg), cow urine (1 L), and jaggery (100 g) in 10 L of water within an earthen pot. DOGROF-2: The formulation was prepared by mixing gram flour (1 kg), crushed *Calotropis* leaves (1 kg), neem leaves (1 kg), cow dung (1 kg), cow urine (1 L), jaggery (100 g), virgin soil (7.25 g), and lime (200 g) in 10 L of clean water in a similar earthen pot. DOGROF-3: The formulation was prepared by mixing bajra flour (1 kg), crushed *Calotropis* leaves (1 kg), karanj leaves (1 kg), ginger powder (50 g), turmeric powder (50 g), and asafoetida (hing) powder (10 g) in 10 L of water in an earthen pot. The mixture was stirred thoroughly to ensure uniformity in earthen pots, covered, and kept under shade with the pots partially buried in soil for 15 days to allow natural fermentation. The contents were periodically stirred to maintain homogeneity throughout the process. After fermentation, the liquid was filtered through muslin cloth and the filtrate was used for foliar spraying on the onion crop. DOGROF-4: This formulation was prepared by mixing neem leaves (500 g), custard apple leaves (500 g), *Calotropis* leaves (500 g), garlic (250 g), and chilli powder (250 g) in 5 L of cow urine. The mixture was stirred thoroughly to ensure homogeneity and used immediately for foliar spraying without fermentation.

### 2.2 Enumeration of microbes in organic formulations

Filtered extracts of the organic formulations were subjected to microbial enumeration to determine the viable microbial population. Appropriate serial dilutions were prepared and plated using standard spread plate techniques (Subbarao, 1999). Nutrient Agar (NA) was used as non-selective medium that supports the growth of a wide range of heterotrophic bacteria, and Potato Dextrose Agar (PDA) was used for general fungal population because it is a nutrient-rich medium promote rapid fungal growth and restricts bacterial growth due to higher pH. Rose Bengal Agar (RBA) was employed as a semi-selective medium to restrict the spread of fast-growing fungi and enable accurate enumeration, while Czapek Dox Agar (CZA), a defined medium with nitrate as the nitrogen source, was used to support growth of actinomycetes. The population was expressed as colony-forming units per mL (cfu/mL) of bioformulation.

### 2.3 Nutrient analysis of organic formulations

Application-ready organic formulations (DOGROF-1 to DOGROF-4) were used for nutrient analysis. Total nitrogen in the organic formulations was estimated by the micro-Kjeldahl method using a Kjeldahl Digestion Unit (Model: 12–0606 Turbotherm Digestion Unit, Gerhardt, Germany) and a Distillation Unit (Model: VAPODEST 200, Gerhardt, Germany). For elemental analysis, the samples were digested using a di-acid mixture (HNO₃:HClO>₄ in a 9:4 ratio). After complete digestion, the digests were diluted with distilled water and filtered through Whatman No. 42 filter paper. The filtrates were subsequently used for the estimation of total N, P, K, S, and micronutrient cations (Fe, Mn, Zn, and Cu). Total P was quantified using the vanadomolybdate yellow color method, and absorbance was recorded at 420 nm using a UV-Visible Spectrophotometer (Model: Spectrostar Nano, BMG Labtech, Germany). Total S was estimated by the turbidimetric method, and absorbance was measured at 440 nm using the same instrument. Total K was determined using a microcontroller-based flame photometer (Model: LT-6715, Labtronics, India). Micronutrient cations were analyzed using an Atomic Absorption Spectrophotometer (Model: PinAAcle 500, PerkinElmer, Singapore).

### 2.4 LC-MS analysis of non-volatile/semi- volatile compounds of organic formulations

High-resolution LC/MS analysis was performed using Agilent Q-ToF G6540B Mass Spectrometry system. One mL of homogenized sample of organic formulation was extracted with 10 mL of acetone–water (70:30, v/v) containing 0.1% formic acid. The mixture was sonicated for 30 min at room temperature and agitated on orbital shaker for 12 h, and then centrifuged at 10,000 rpm for 10 min. The supernatant was collected and filtered through a 0.22 μm PTFE membrane filter. The acetone–water extract was evaporated to dryness and the residue was reconstituted in LC grade methanol–water (50:50, v/v) for analysis. The metabolite separation was performed with the high-performance liquid chromatography system coupled with Agilent Eclipse XDB-C18, 3X150 mm (Internal Diameter: 3.0 mm, Length: 150 mm, Particle size 3.5 micron) column. The mobile phases were prepared with HPLC-grade deionized water with 0.1% formic acid (Solvent A) and acetonitrile with formic acid 0.1% (Solvent B). The elution gradient program was started with time-A/B: 0 min-95/5, 2 min-95/5, 25 min-5/95, 28 min-5/95, 28.1 min-95/5, 30 min-95/5. The injection volume of the sample was set at 15 µL, at a flow rate of 0.4 mL min⁻¹ and the column temperature was 40 °C. The mass spectrometric analysis was performed with a Dual AJS ESI source (MS Scan Range: at *m/z* 100 to 1700) and Ion Source Parameters were Nebulizer Gas Temp: 300 °C; Sheath Gas Temp: 350 °C; Drying Gas: 8 l/min; Nebulizer Gas: 35 psig; Sheath Gas Flow: 11 l/min; Capillary voltage: 3500 V; Nozzle Voltage: 1000 V. Each sample was analyzed in positive mode. Raw data files (.d format) were processed using Agilent MassHunter Qualitative Analysis software (version 10.0). Molecular features were extracted using the Molecular Feature Extraction (MFE) algorithm and compound annotation was performed based on m/z matching with a mass tolerance of ±5 ppm against the METLIN Metabolites Personal Compound Database and Library (PCDL, B.08.00). Due to the absence of MS/MS-based structural confirmation and validation with authentic standards, the reported metabolites are considered putative identifications.

### 2.5 Study site and field evaluation of organic formulations

#### 2.5.1 Study site description

The study was conducted at the experimental research farm of ICAR–Directorate of Onion and Garlic Research, Pune, Maharashtra, India, located at Rajgurunagar (18.5035° N, 73.5305° E; 611 m above mean sea level). The experimental site lies on the western part of the Deccan Plateau and is characterized by a semi-arid tropical climate with a mean annual rainfall ranging between 650–700 mm, predominantly received during June–September. The monthly mean minimum temperatures at the site were 10.5 °C, 11.7 °C, and 11.7 °C, whereas the corresponding monthly mean maximum temperatures were 37.5 °C, 39.0 °C, and 37.4 °C during 2021, 2022, and 2023, respectively.

#### 2.5.2 Experimental design

The field experiment was laid out in a Randomized Block Design (RBD) with seven treatments and three replications. Each experimental plot measured 2 m × 3 m. Field experiments were conducted during *Kharif* and *Rabi* season for three consecutive years (2021-2023). Popular recommended onion varieties, *viz*. Bhima Super (*Kharif*) and Bhima Shakti (*Rabi*), developed by ICAR-DOGR, were used to evaluate the efficacy of bioformulations in comparison with existing practices, farmer practices, and an untreated control under natural field conditions. Organic formulations were applied as foliar sprays at a concentration of 125 mL/10L (Raskar and Wani, 2014) at 30, 45, 60, and 75 days after transplanting (DAT). The experiment also included a recommended Existing Practice (EP) and a Farmer Practice (FP) for comparison. In the EP treatment, seed treatment was done with carbendazim (2 g/kg seed), followed by dipping of seedlings in solution of prophenophos (1 mL/L) and carbendazim (1 g/L) before transplanting. Subsequent sprays included mancozeb (2.5 g/L) at 30 DAT; a combination of prophenophos (1 mL/L) and tricyclazole (1 mL/L) at 45 DAT; and prophenophos (1 mL/L) at 60 and 70 DAT. The FP treatment involved spraying carbendazim (2 g/L) or mancozeb (2.5 g/L) at 15-day intervals, specifically at 15, 30, 45, 60, 75, and 90 DAT. A basal mineral fertilizer dose of 100:50:50:30 kg/ha N:P:K:S was applied uniformly to all plots, and other standard agronomic practices were followed throughout the crop growth period. Ten plants in each plot were scored for disease observation by using a 0–5 disease rating scale for Stemphylium blight (0- No disease; 1-10%; 2-11-20%; 3-21-30%; 4-31-50%; 5-51-100%) as detailed in **Supplementary Table S1**. Anthracnose-twister disease severity was assessed using the 0–9 grade scale (**Supplementary Table S2**) for scoring the disease (Dutta et al., 2022), and the Percent Disease Index (PDI) was calculated as described by Wheeler (1969). At 60 days after treatment application, the crop growth parameters, *viz*., number of leaves, plant height (cm), and pseudostem diameter (mm), were recorded. The bulb yield per plot was expressed in t/ha.

#### 2.5.3 ICBR calculation

The incremental cost benefit ratio (ICBR) was calculated for all treatments using the formula described by Chejara (2013), *i.e*., ICBR=Additional income received (from the particular treatment)/Additional cost incurred for the particular treatment. The additional income and cost were estimated based on the prevailing market rates. The storability of *Kharif* onion is comparatively low; hence, the produce is marketed immediately after harvest. Accordingly, the income for *Kharif* onion was calculated based on a market price of 20/kg. For *Rabi* onion, the ICBR was calculated considering the prevailing price during May (harvest period), when market rates are considerably low (7/kg). Additionally, to assess the potential benefit of extended storage, an alternative ICBR was calculated for *Rabi* produce sold during September-October, when market prices rise to approximately 20/kg. While estimating the post-storage returns, a minimum storability loss of 15% was factored into the calculation to account for quantitative and qualitative losses during storage.

### 2.6 Statistical analysis

All experimental data and derived variables were subjected to statistical analysis to assess the effects of different bioformulations on crop growth, yield, and disease parameters recorded over three consecutive years (2021–2023). For multi-season trials, pooled analysis across years was conducted after confirming the homogeneity of error variances. Treatments were considered fixed effects, whereas year was treated as a random effect in the pooled analysis. Treatment means were compared using the Least Significant Difference (LSD) test at a significance level of *p* ≤ 0.05, following a significant F-test. Interaction effects between treatments and years were also evaluated to assess the consistency of treatment performance across seasons. All statistical analyses were carried out using SPSS Statistics Version 22.0 (IBM Corp., Armonk, NY, USA).

## 3 Results

### 3.1 Microbial populations in organic formulations

Microbial enumeration of four organic formulations (DOGROF-1 to DOGROF-4) revealed significant differences in total viable counts (**Table 1**). DOGROF-3 exhibited the highest microbial load, with bacteria at 3 × 10^8^ cfu/mL, fungi at 4 × 10^5^ cfu/mL and actinomycetes at 8 × 10^5^ cfu/mL. DOGROF-1 and DOGROF-2 showed comparable bacterial (1 × 10^8^ cfu/mL) and fungal (1 × 10^5^ cfu/mL) counts; however, DOGROF-2 contained a higher actinomycete population (3 × 10^5^ cfu/mL) than DOGROF-1 (1 × 10^5^ cfu/mL). DOGROF-4 also supported substantial microbial populations, with bacterial, fungal, and actinomycete counts of 2 × 10^8^, 1 × 10^5^, and 2 × 10^5^ cfu/mL, respectively. These results indicate that DOGROF-3 is the most microbiologically active formulation, followed by DOGROF-4, DOGROF-2 and DOGROF-1.

**Table 1:** Bacterial, fungal and actinomycetes CFU counts in four bioformulations (DOGROF-1 to DOGROF-4).

Treatments	Bacteria (cfu/mL)	Fungi (cfu/mL)	Actinomycetes (cfu/mL)
DOGROF-1	1 × 10^8^	1 × 10^5^	1 × 10^5^
DOGROF-2	1 × 10^8^	1 × 10^5^	3 × 10^5^
DOGROF-3	3 × 10^8^	4 × 10^5^	8 × 10^5^
DOGROF-4	2 × 10^8^	1 × 10^5^	2 × 10^5^

### 3.2 Nutrient composition of formulations

The nutrient profiles of organic formulations DOGROF-1-DOGROF-4 showed large variation in both macro and micronutrient status (**Table 2**). Nitrogen content ranged from 0.09% in DOGROF-3 to 0.20% in DOGROF-2. Phosphorus was lowest in DOGROF-3 (0.97%) and highest in DOGROF-4 (2.37%), while potassium ranged from 3.42% in DOGROF-3 to 23.59% in DOGROF-4. Sulphur concentration was moderate across formulations, with DOGROF-4 showing the highest level (2.76%). In contrast, micronutrient concentrations differed markedly among formulations. DOGROF-3 contained substantially higher zinc (20.25 ppm), manganese (8.62 ppm) and copper (7.18 ppm) than DOGROF-1 and DOGROF-2. Iron content in DOGROF-3 (2.42 ppm) was higher than DOGROF-1 and comparable with DOGROF-2, but much lower than DOGROF-4. Despite its lower macronutrient content, particularly nitrogen and phosphorus, DOGROF-3 recorded superior onion yield compared with DOGROF-1 and DOGROF-2, indicating that crop response was not governed solely by nutrient quantity.

**Table 2:** Macro- and micronutrient composition of four bioformulations (DOGROF-1–DOGROF-4).

Nutrients	Nutrient status			
	DOGROF-1	DOGROF-2	DOGROF-3	DOGROF-4
Nitrogen (%)	0.19 ± 0.002	0.20 ± 0.003	0.09 ± 0.002	0.15 ± 0.018
Phosphorous (%)	1.69 ± 0.008	1.76 ± 0.022	0.97 ± 0.035	2.37 ± 0.118
Potassium (%)	7.79 ± 0.043	10.00 ± 0.091	3.42 ± 0.045	23.59 ± 0.541
Sulphur (%)	1.23 ± 0.019	0.88 ± 0.007	0.82 ± 0.023	2.76 ± 0.008
Iron (ppm)	0.49 ± 0.005	3.12 ± 0.005	2.42 ± 0.268	26.86 ± 0.576
Manganese (ppm)	0.39 ± 0.023	3.10 ± 0.087	8.62 ± 0.103	55.17 ± 0.061
Zinc (ppm)	1.02 ± 0.020	3.47 ± 0.055	20.25 ± 0.188	68.24 ± 0.301
Copper (ppm)	0.56 ± 0.009	3.17 ± 0.010	7.18 ± 0.071	50.75 ± 0.531

### 3.3 Non-volatile metabolite profiles of organic formulations

**Figure 1:**
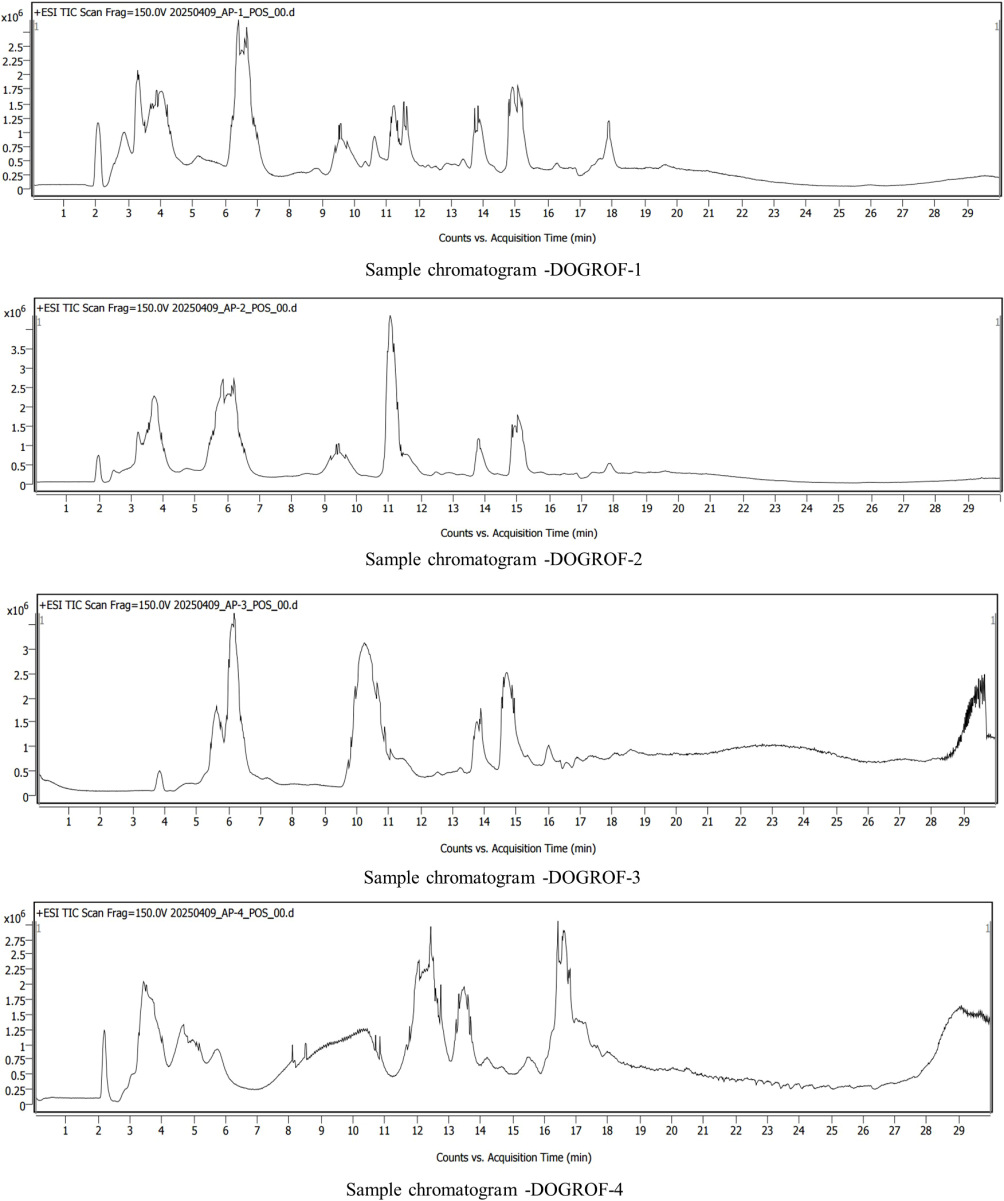
The representative TIC (Total Ion Chromatogram) of organic formulations (DOGROF-1 to DOGROF-4) obtained by LC–ESI–MS analysis in positive ionization mode (+ESI) showing the acquisition time on x-axis and ion intensity on y-axis for the analyzed samples.

Liquid chromatography-mass spectrometry (LC-MS) profiling of four organic formulations (DOGROF-1 to DOGROF-4) revealed marked variation in their non-volatile chemical composition, as summarized in **Table 3**. To ensure annotation confidence, **Table 3** includes only those compounds that met stringent filtering criteria of database match score greater than 90 and the availability of valid compound names and CAS (Chemical Abstracts Service) registry numbers while metabolic features annotated solely at the molecular formula level, without valid compound names, were excluded. The LC chromatograms of all the formulations is depicted on **Figure 1**. The putatively identified non-volatile compounds detected across the DOGROF-1 included 3-dehydro-L-threonate, threonate, altretamine, caffeoyl alcohol, 2-methylbenzaldehyde, swietenidin B, boschniakine, (R)-1-O-[β-D-glucopyranosyl-(1→6)-β-D-glucopyranoside]-1,3-octanediol, epothilone D, degraded cyanogenic glycosides of Sambucus nigra (2′-epimer), and decarbamoylneosaxitoxin whereas the putatively identified compounds in DOGROF-2 included 3-Hydroxyisoheptanoic acid, 1-(4′-Hydroxyphenyl) ethanol, 2-Methylbenzaldehyde, Levonordefrin, L-isoleucyl-L-proline, 6-Deoxyerythronolide B, Boschniakine, Swietenidin B, Epothilone D, and Triethyl citrate. Comparative analysis of the LC–MS profiles revealed that DOGROF-1 and DOGROF-2 shared four compounds putatively identified as 2-Methylbenzaldehyde, Swietenidin B, Boschniakine, and Epothilone D, indicating partial overlap in their non-volatile chemical composition. In contrast, DOGROF-3 and DOGROF-4 exhibited distinct sets of putatively annotated compounds, reflecting formulation-specific non-volatile profiles.

**Table 3:** LC–MS-based identification of putative compounds in four different organic formulations (DOGROF-1 to DOGROF-4), showing retention time (RT), m/z ratio, adduct/species, calculated neutral mass, and putative chemical formula of each detected compound.

Retention Time (RT)	m/z ratio	Adduct/species	Calculated neutral mass	Putative chemical formula	Putative identified compound
DOGROF-1					
3.299	152.0559	(M+NH_4_)^+^	134.0221	C_4_H_6_O_5_	3-Dehydro-L-threonate
3.429	233.1480	(M+Na)^+^	210.1588	C_9_H_18_N_6_	Altretamine
3.602	137.0450	(M+H)^+^	136.0378	C_4_H_8_O_5_	Threonate
6.533	166.0864	(M+NH_4_)^+^[-H_2_O]	166.0630	C_9_H_10_O_3_	Caffeyl alcohol
6.545	120.0804	(M+NH_4_)^+^[-H_2_O]	120.0571	C_8_H_8_O	2-Methylbenzaldehyde
10.638	188.0699	(M+H)^+^[-H_2_O]	205.0731	C_11_H_11_NO_3_	Swietenidin B
11.242	144.0812	(M+H)^+^[-H_2_O]	161.0844	C_10_H_11_NO	Boschniakine
12.304	488.2716	(M+NH_4_)^+^	470.2375	C_20_H_38_O_12_	(R)-1-O-[b-D-Glucopyranosyl-(1->6)-b-D-glucopyranoside]-1,3-octanediol
13.926	491.2944	(M+NH_4_)^+^[-H_2_O]	491.2708	C_27_H_41_NO_5_S	Epothilone D
17.227	371.1454	(M+NH_4_)^+^	353.1116	C_16_H_19_NO_8_	Sambucus nigra Degraded cyanogenic glycosides (2'-Epimer)
19.629	290.1574	(M+NH_4_)^+^	272.1236	C_9_H_16_N_6_O_4_	Decarbamoylneosaxitoxin
DOGROF-2					
3.112	146.1169	(M+NH_4_)^+^[-H_2_O]	146.0936	C_7_H_14_O_3_	3-Hydroxyisoheptanoic acid
3.737	121.0647	(M+H)^+^[-H_2_O]	138.0679	C_8_H_10_O_2_	1-(4'-Hydroxyphenyl)ethanol
5.992	120.0805	(M+NH_4_)^+^[-H_2_O]	120.0573	C_8_H_8_O	2-Methylbenzaldehyde
6.006	166.0864	(M+H)^+^[-H_2_O]	183.0896	C_9_H_13_NO_3_	Levonordefrin
9.424	229.1548	(M+H)^+^	228.1476	C_11_H_20_N_2_O_3_	L-isoleucyl-L-proline
11.000	387.2736	(M+H)^+^	386.2661	C_21_H_38_O_6_	6-Deoxyerythronolide B
11.077	144.0806	(M+H)^+^[-H_2_O]	161.0839	C_10_H_11_NO	Boschniakine
11.639	188.0700	(M+H)^+^[-H_2_O]	205.0732	C_11_H_11_NO_3_	Swietenidin B
13.831	491.2950	(M+NH_4_)^+^[-H_2_O]	491.2716	C_27_H_41_NO_5_S	Epothilone D
17.864	276.1435	(M+NH_4_)^+^[-H_2_O]	276.1202	C_12_H_20_O_7_	Triethyl citrate
DOGROF-3					
6.194	392.1846	(M+H)^+^	391.1775	C_16_H_29_N_3_O_6_S	Hexylglutathione
11.415	188.0734	(M+NH_4_)^+^	170.0397	C_8_H_10_O_2_S	2,5-Dimethyl-3-furanthiol acetate
15.106	619.2778	(M+Na)^+^	596.2885	C_36_H_40_N_2_O_6_	Guattegaumerine
17.502	643.2741	(M+Na)^+^	620.2848	C_32_H_44_O_12_	Lanceotoxin A
22.274	555.2956	(M+H)^+^[-H_2_O]	572.2986	C_32_H_44_O_9_	Ganoderic acid H
DOGROF-4					
3.625	174.1257	(M+Na)^+^[-H_2_O]	169.1469	C_10_H_19_NO	Lupinine
5.794	310.1311	(M+Na)^+^[-H_2_O]	305.1525	C_11_H_23_N_5_O_3_S	Arginyl-Methionine
13.473	295.1331	(M+H)^+^[-H_2_O]	312.1361	C_19_H_20_O_4_	m-(beta-Acetyl-alpha-ethyl-p-hydroxyphenethyl)benzoic acid
14.126	310.1463	(M+H)^+^	309.1390	C_17_H_16_FN_5_	SB 218655
14.508	380.0968	(M+H)^+^	379.0894	C_10_H_17_N_7_O_7_S	Gonyautoxin 5
15.760	476.3082	(M+H)^+^	475.3008	C_21_H_41_N_5_O_7_	Netilmicin
16.343	625.1767	(M+H)^+^	624.1693	C_28_H_32_O_16_	Isorhamnetin 3-O-[b-D-glucopyranosyl-(1->2)-a-L-rhamnopyranoside]
16.834	678.3695	(M+NH_4_)^+^	660.3356	C_32_H_52_O_14_	Capsianoside I
18.138	527.2642	(M+H)^+^[-H_2_O]	544.2674	C_30_H_40_O_9_	Physagulin F
19.601	515.2645	(M+H)^+^[-H_2_O]	532.2677	C_29_H_40_O_9_	Roridin A
19.747	574.3008	(M+NH_4_)^+^[-H_2_O]	574.2772	C_31_H_42_O_10_	Asclepin
20.483	1131.5601	(M+Na)^+^	1108.5705	C_59_H_84_N_2_O_18_	Candicidin D

The putatively annotated compounds detected in DOGROF-3 included Hexylglutathione, 2,5-Dimethyl-3-furanthiol acetate, Guattegaumerine, Lanceotoxin A, and Ganoderic acid H whereas the putatively identified compounds in DOGROF-4 included Lupinine, Arginyl-Methionine, m-(beta-Acetyl-alpha-ethyl-p-hydroxyphenethyl)benzoic acid, SB 218655, Gonyautoxin 5, Netilmicin, Isorhamnetin 3-O-[b-D-glucopyranosyl-(1->2)-a-L-rhamnopyranoside], Capsianoside I, Physagulin F, Roridin A, Asclepin, and Candicidin D. Collectively, the LC–MS data highlighted a substantial heterogeneity in the non-volatile chemical composition of the four bioformulations. The coexistence of phenolic acids, indolic compounds, and sulfur-containing non-volatiles suggests that these formulations possess multifaceted bioactivity.

### 3.4 Effect of organic formulations on vegetative growth of onion

The pooled data for three consecutive years (2021–2023) revealed significant differences (p ≤ 0.05) among treatments for all vegetative growth parameters, including plant height, pseudostem diameter, and number of leaves per plant in both *Kharif* and *Rabi* seasons (**Table 4**; **Supplementary Figures S1, S2**). During the *Kharif* season, plant height varied from 38.42 cm to 48.52 cm, with the highest value (48.52 cm) recorded in DOGROF-3, which was statistically superior to all other treatments. Treatments DOGROF-4 (45.09 cm) and DOGROF-2 (43.15 cm) also recorded significantly higher plant height compared to the control, whereas EP and FP remained statistically at par with each other. A similar trend was observed for pseudostem diameter, which ranged from 9.41 mm to 13.09 mm. Treatment DOGROF-3 showed the thickest (13.09 mm) pseudostem, significantly higher than all other treatments, followed by DOGROF-4 (12.23 mm) and EP (11.44 mm). The minimum pseudostem diameter in the control (9.41mm) reflected reduced vigor under untreated conditions. The number of leaves per plant followed a similar pattern, ranging from 4.91 to 6.89. DOGROF-3 also produced the maximum number of leaves, followed by DOGROF-4 (6.51) and EP (6.29), while the lowest leaf count was observed in the untreated control. The LSD for treatments indicated significant separation (p ≤ 0.05) among the means for all three parameters, confirming strong treatment effects on vegetative growth during the *Kharif* season.

**Table 4:** Effect of application of organic formulations on the crop health of onion (pooled mean of *Kharif* and *Rabi* 2021-2023).

Treatments	*Kharif* 2021-2023			*Rabi* 2021-2023		
	Plant height (cm)	No. of leaves/plant	Pseudostem diameter (mm)	Plant height (cm)	No. of leaves/plant	Pseudostem diameter (mm)
DOGROF-1	41.38^bc ±^1.72*	6.00 ^bc ±^0.20	11.32^c ±^0.30	50.97^a^ ± 2.07	7.98 ^bc ±^0.22	14.51^a ±^0.23
DOGROF-2	43.15^b ±^4.23	6.04 ^bc ±^0.25	11.18^c ±^0.69	50.77^a ±^1.72	7.87 ^bc ±^0.17	14.59^a ±^0.19
DOGROF-3	48.52^a ±^2.17	6.89 ^a ±^0.25	13.09^a ±^0.27	53.40^a ±^1.20	8.64 ^a ±^0.12	15.00^a ±^0.27
DOGROF-4	45.09 ^ab ±^1.18	6.51^ab ±^0.21	12.23^b ±^0.52	52.01^a ±^1.47	8.20 ^ab ±^0.10	14.66^a ±^0.40
Existing practice	42.52^bc ±^1.20	6.29^ab ±^0.07	11.44^bc ±^0.20	44.64^b ±^2.35	8.00 ^bc ±^0.04	13.89 ^ab ±^0.67
Farmers Practice	43.19^b ±^2.37	5.51^cd ±^0.38	10.61^c ±^0.72	43.04^b ±^1.70	7.56 ^c ±^0.23	12.97^b ±^0.31
Untreated control	38.42^a ±^1.08	4.91^d ±^0.82	9.41^d ±^0.37	39.09^c ±^0.20	6.27 ^d ±^0.03	10.89^c ±^0.09
LSD (p ≤ 0.05) (Treatments)	3.06	0.81	1.24	2.16	0.48	0.96
LSD (p ≤ 0.05) (Year)	2.01	0.53	0.81	1.42	0.32	0.63
LSD (p ≤ 0.05) (Treatments ×Years)	5.31	1.40	2.14	3.75	0.84	1.66

*Values represent mean ± standard deviation (SD) of three replications. Means followed by the same letter within a column are not significantly different according to the least significant difference (LSD) test at p ≤ 0.05.

In the *Rabi* season, the results showed a consistent trend with overall higher vegetative growth than in *Kharif*. Plant height ranged from 39.09 cm to 53.40 cm, with DOGROF-3 (53.40 cm) and DOGROF-4 (52.01 cm) statistically at par and superior to all other treatments. The pseudostem diameter was significantly greater in DOGROF-3 (15.00 mm), which was at par with DOGROF-4 (14.66 mm), DOGROF-2 (14.59 mm) and DOGROF-1 (14.51 mm), while the minimum value (10.89 mm) was observed in the untreated control. The number of leaves per plant ranged from 6.27 to 8.64. The LSD values indicated that the treatment effects were significant for all traits, with lesser variation across years, suggesting the reproducibility and stability of responses across the three-year evaluation. The interaction effects between treatments and years were also significant, confirming the consistency of treatment performance over multiple seasons and years. Overall, the DOGROF-3 formulation consistently recorded the highest values for all growth parameters across both seasons 2021-23, followed by DOGROF-4 and DOGROF-2 as compared to EP and FP. The untreated control consistently recorded the lowest growth performance (The year wise data presented in **Supplementary Tables S3**, **S4**).

### 3.5 Effect of organic formulations on diseases and yield performance of onion

The field efficacy of organic formulations against anthracnose-twister and Stemphylium blight of onion was assessed over three consecutive *Kharif* and *Rabi* seasons (2021-2023) (**Supplementary Tables S5**, **S6**), and the pooled analysis results are presented in **Table 5** and supplementary **Supplementary Figures S3**, **S4**. Significant differences were observed among treatments in reducing the severity of Anthracnose-twister and Stemphylium blight diseases. The application of organic formulations markedly influenced the yield improvement, and cost-benefit efficiency compared with the untreated control across three consecutive years, with the highest yield (20.21 t/ha *Kharif* and 29. 76t/ha *Rabi*) recorded in 2021 for DOGROF-3, whereas the control plots produced only 13.11 t/ha in *Kharif* and 18.55 t/ha in *Rabi* in the same year.

**Table 5:** Effect of application of organic formulations on Anthracnose-twister and Stemphylium blight disease severity on onion (pooled mean of *Kharif* and *Rabi* 2021-2023).

Treatments	Kharif 2021-2023					Rabi 2021-2023				
	Anthracnose-twister disease severity (%)	Percent disease reduction over Control	Yield (t/ha)	Yield increase (%)	ICBR	Stemphylium blight	Percent disease reduction over Control	Yield (t/ha)	Yield increase (%)	ICBR
DOGROF-1	38.30^b^ ±6.93	38.77	16.19 ^abc^ ±1.81	19.69	20.54	44.33^c^ ±3.17	27.32	23.18 ^b^ ±0.29	8.10	23.50
DOGROF-2	41.06^bc^ ±7.62	34.35	15.92 ^abc^ ±0.85	17.68	18.72	48.00^b^ ±1.52	21.31	24.49 ^b^ ±1.01	14.18	26.54
DOGROF-3	31.51^a^ ± 4.42	49.62	18.16 ^a^ ±1.08	34.23	33.65	31.67^a^ ±1.20	48.09	27.70 ^a^ ±1.20	29.17	34.04
DOGROF-4	38.15^b^ ±7.19	39.01	16.57 ^ab^ ±0.51	22.52	23.10	36.33^a^ ±3.28	40.44	24.03 ^b^ ±0.93	12.03	25.50
Existing practice	42.88^bc^ ±4.16	31.45	15.46 ^abc^ ±2.52	14.26	4.70	44.47^c^ ±2.62	27.09	24.32 ^b^ ±0.79	13.40	7.85
Farmers Practice	47.53^c^ ±6.26	24.00	14.74 ^bc^ ±1.73	9.00	3.30	44.97^c^ ± 2.02	26.28	22.91 ^b^ ±0.78	6.85	6.90
Untreated control	62.54^d^ ± 2.71	–	13.53 ^c^ ±0.42	–	0.0	61.00 ^d^ ±2.0	–	21.44 ^b^ ±1.05	–	0.0
LSD (p ≤ 0.05) (Treatments)	1.53		1.57			1.84		1.57		
LSD (p ≤ 0.05) (Year)	2.34		2.40			3.45		2.40		
LSD (p ≤ 0.05) (Treatments ×Years)	4.06		4.16			4.87		4.16		

*Values represent mean ± standard deviation (SD) of three replications. Means followed by the same letter within a column are not significantly different according to the least significant difference (LSD) test at p ≤ 0.05.

During the *Kharif* season, the lowest Anthracnose-twister disease severity (31.51%) was recorded in DOGROF-3, which corresponded to the highest percent disease control of 49.62%. This treatment also registered a significantly higher bulb yield (18.16 t/ha), reflecting a 34.23% increase over the untreated control. The next best performance was observed in DOGROF-4, showing 38.15% disease severity with 39.01% PDC and a 22.52% yield increase. In contrast, the untreated control recorded the highest disease severity (62.54%) and the lowest yield (13.53 t/ha). The ICBR was maximum (33.65) for DOGROF-3, followed by DOGROF-4 (23.10).

Similarly, during the *Rabi* season, DOGROF-3 remained the most effective, significantly reducing Stemphylium blight severity to 31.67% with 48.09% PDC. This treatment produced the highest yield (27.70 t/ha), representing a 29.17% increase over the control (21.44 t/ha), and achieved the highest ICBR (34.04). DOGROF-4 also performed well, reducing disease severity to 36.33% (40.44% PDC) and increasing yield by 12.03% over the control. The conventional farmer’s practice (FP) and chemical-based practice (EP) showed moderate disease suppression and yield gains, while the untreated control consistently exhibited the highest disease incidence and lowest productivity across both seasons.

Overall, the pooled data from 2021–2023 clearly demonstrate that DOGROF-3 was the most effective organic formulation in minimizing disease severity and maximizing onion yield, followed by DOGROF-4. These results suggest that the synergistic combination of plant-derived bioactives and microbial metabolites in DOGROF-3 played a crucial role in enhancing plant health and suppressing foliar pathogens under field conditions.

## 4 Discussion

Organic formulations prepared from locally available natural resources represent an emerging promising strategy for managing soil fertility and plant health in onion production systems. In the present study, distinct differences were observed among the four organic bioformulations (DOGROF-1 to DOGROF-4) in terms of their microbial populations, nutrient composition, metabolite profile and efficacy in reducing foliar disease incidence in onion. The microbial enumeration results indicated that DOGROF-3 supported the highest bacterial and actinomycete populations, demonstrating that the substrate composition in this formulation was more conducive to the proliferation of beneficial microflora. The superior microbial enrichment observed in DOGROF-3 could be attributed to the balanced carbon and nitrogen sources provided by gram and bajra flour which favor the growth of heterotrophic bacteria and actinomycetes actively involved in organic matter decomposition and nutrient mineralization (Sharma et al., 2020). Although DOGROF-1 and DOGROF-2 exhibited comparable bacterial and fungal populations, DOGROF-2 supported a relatively higher actinomycete load than DOGROF-1, indicating formulation-specific differences that may favor the proliferation of certain microbial groups. Despite the absence of a fermentation process in its preparation, DOGROF-4 sustained appreciable populations of bacteria, fungi, and actinomycetes comparable to DOGROF-1 and DOGROF-2. In contrast, DOGROF-3 emerged as the most conducive formulation for microbial growth, supporting the highest CFU counts across all microbial groups. These variations highlight the influence of formulation composition on microbial survival and proliferation (Deori et al., 2025). Similarly, the fungal population was markedly higher in DOGROF-3, which could be due to the presence of readily fermentable carbon sources and decomposable organic residues that promote fungal colonization and metabolic activity. The reduced fungal populations in DOGROF-1, DOGROF-2 and DOGROF-4 are likely associated with the antimicrobial properties of neem leaf extracts included in these formulations. These botanical amendments contain bioactive compounds such as azadirachtin, uscharin, and calotropin that are known to exhibit strong antifungal and antibacterial effects (Usha et al., 2009; Kumar and Neeraj, 2015; Singh et al., 2019). In addition, the higher actinomycetes population observed in DOGROF-3 may have contributed to enhanced growth of onion crop. Actinomycetes are well documented for their ability to produce phytohormones, solubilize minerals, and act as biocontrol agents against phytopathogenic fungi (Mitra et al., 2022; Torres-Rodriguez et al., 2022). Their antagonistic activity is mediated by competition for space and nutrients, production of polyene antibiotics, inhibition of fungal cell wall biosynthesis, secretion of extracellular lytic enzymes such as chitinases, and induction of plant defense responses (Lahdenpera et al., 1991; Kabaluk et al., 2010; Copping and Duke, 2007). Collectively, these findings corroborate earlier reports that organic formulations enriched with diverse microbial consortia enhance soil microbial diversity and contribute to the suppression of soil and foliar-borne pathogens, thereby improving plant health and disease resilience (Bhattacharjya et al., 2021).

Traditional organic formulations play a vital role in promoting crop growth through their rich microbial and metabolic profiles. For instance, formulations like Amritpani are rich in plant growth-promoting bacteria such as *Bacillus, Enterobacter, Pseudomonas, Azospirillum, Agrobacterium*, and *Rhizobium*. These microbes produce indole-3-acetic acid (IAA), which enhances cell division, stimulates root development, and activates the plant’s defense systems (Kumar et al., 2021; Nandhini and Somasundaram, 2023). Collectively, the higher microbial load observed in DOGROF-1 and DOGROF-3, as evidenced by CFU counts, suggests their potential as effective organic formulations. The elevated viable microbial population may enhance microbe-mediated nutrient transformations in soil, which could in turn support plant growth and possibly improve disease tolerance in onion under field conditions. However, the results are only indicative, given the inherent limitations of CFU-based approaches, including their bias toward culturable microorganisms, media-dependent selectivity, and variability associated with plating and colony development. Therefore, the further investigations involving culture-independent metagenomics approaches to decipher the detailed community structure and function would be required to substantiate these functional implications.

The higher yield obtained with DOGROF-3, despite its lower nitrogen and phosphorus content, suggests that nutrient balance and bioavailability were more important than total nutrient concentration (Marschner, 2012). The superior performance of DOGROF-3 might be attributed to its enriched micronutrient status, especially zinc, manganese and copper. Zinc plays an essential role in enzyme activation, auxin production and carbohydrate metabolism, all of which strongly influence bulb development in onion (Alloway, 2008; Cakmak, 2008). The higher zinc concentration in DOGROF-3 might have enhanced nitrogen utilization efficiency and assimilate partitioning to bulbs. Manganese in DOGROF-3 may have improved photosynthetic efficiency through its role in photosystem II and enzymatic regulation, while copper may have supported reproductive growth and carbohydrate transport. Additionally, the ingredients used in DOGROF-3 (bajra flour, *Calotropis*, Karanj, ginger, turmeric and asafoetida) likely exerted indirect biological effects. Bajra flour likely served as a carbon source that may have stimulated the proliferation of viable microbial populations, potentially enhancing nutrient mineralization and improving root nutrient availability (Kuzyakov and Blagodatskaya, 2015). The phytochemicals present in ginger, turmeric and asafoetida are known to possess antimicrobial properties and may have suppressed disease incidence, contributing to improved plant health and yield (Singh et al., 2002; Jayaprakasha et al., 2005; Prasad and Aggarwal, 2011; Kalhoro et al., 2022). In contrast, DOGROF-1 and DOGROF-2, although higher in NPK, were deficient in key micronutrients. Furthermore, lime and virgin soil in DOGROF-2 may have restricted micronutrient availability through pH modification. These factors likely limited the physiological efficiency of onion plants despite adequate macronutrients (Lindsay, 1979; Marschner, 2012). Although DOGROF-4 contained the higher concentrations of N, P, K, and micronutrients, its reduced effectiveness in enhancing onion growth, yield, and disease suppression may be attributed to nutrient imbalance and phytotoxic effects. Excess potassium can antagonize the uptake of calcium and magnesium, while elevated levels of iron, zinc, and copper may interfere with metabolic processes and may induce oxidative stress, thereby reducing nutrient use efficiency (Kabata-Pendias and Pendias, 2001; Broadley et al., 2012). In addition, the high concentration of bioactive compounds derived from neem, custard apple, *Calotropis*, garlic, and chilli may have exerted allelopathic or phytotoxic effects on onion plants and non-selectively suppressed beneficial rhizosphere microorganisms. Such combined nutritional and biochemical stress likely limited physiological performance and disease resistance in onion despite the apparent abundance of nutrients in DOGROF-4.

LC-MS based metabolite profiling of four organic formulations (DOGROF-1 to DOGROF-4), revealed qualitative differences in their putatively annotated compounds. Although metabolite identities were putatively assigned based on accurate mass matching without MS/MS validation, the comparative metabolite profiles among formulations provided meaningful insights into their potential functional attributes. Among the four formulations used in this study, the DOGROF-3 exhibited a comparatively broader spectrum of putatively identified metabolites including hexylglutathione, 2,5-dimethyl-3-furanthiol acetate, guattegaumerine, lanceotoxin A, and ganoderic acid H. The putative detection of hexylglutathione, a glutathione conjugate, in DOGROF-3 suggests that the efficacy of this bioformulation may be partly associated with enhanced antioxidant potential and oxidative stress mitigation. Glutathione conjugates are central to redox buffering pathways, therefore, their putative presence in DOGROF-3 may have contributed to improved oxidative stress resilience and enhanced physiological stability in treated onion plants. Besides, Glutathione conjugates are integral to glutathione S-transferase (GST)-mediated detoxification pathways, where GSTs catalyze the conjugation of reduced glutathione to reactive metabolites, thereby supporting cellular redox homeostasis. Although GST activity was not directly assessed in this study, the detection of a glutathione-derived conjugate in DOGROF-3 may indicate potential enrichment of glutathione-associated metabolic processes. GSTs are known to participate in oxidative stress regulation, salicylic acid–mediated defense signaling, and induced systemic resistance (ISR) (Gullner et al., 2018). Their protective role has been demonstrated in grapevine, where GST29 reduced esca disease by limiting infection-induced oxidative damage (Mauch and Dudler, 1993; Bertsch et al., 2013). In this context, the putative presence of hexylglutathione may reflect enhanced antioxidant and detoxification capacity, however, in the absence of MS/MS confirmation, these metabolite assignments and their inferred biological roles remain putative and warrant further validations.

The putative detection of alkaloid-type compounds annotated as Guattegaumerine and Lanceotoxin A in DOGROF-3 is of particular interest, as such molecules have been reported to exhibit antimicrobial activity (Leclercq et al., 1987; Zhang et al., 2025). Therefore, the presence of such molecules suggests a possible role in direct pathogen inhibition. Similarly, the putative detection of ganoderic acid H, a triterpenoid-type compound documented for antimicrobial and antioxidant properties, may also partly underlie the disease suppression observed with DOGROF-3 (Li et al., 2012). This biochemical distinction of DOGROF-3 corresponded with its superior field performance, reflected in greater disease reduction against anthracnose-twister and Stemphylium blight, along with yield enhancement across seasons. Therefore, the superior field performance of DOGROF-3, which recorded the higher disease reduction against anthracnose-twister (49.62%) and Stemphylium blight (48.09%), along with greater yield advantages during *Kharif* (34.23%) and *Rabi* (29.17%) seasons, may be associated with the presence of these putatively identified metabolites that were detected exclusively in this formulation. While their precise functional roles require further confirmation through targeted analyses, their unique occurrence in DOGROF-3 suggests a possible contribution to its enhanced disease suppression and yield response.

Furthermore, the metabolite and microbial profiling of the four organic formulations (DOGROF-1 to DOGROF-4) revealed complementary biological functions that collectively explain their differential efficacy in enhancing onion crop health and disease suppression under field conditions. Formulations DOGROF-1 and DOGROF-3 showed a pronounced abundance of beneficial microbes-particularly actinomycetes which are essential for organic matter mineralization and nutrient transformation. This could be attributed to the balanced carbon and nitrogen ratio provided by substrates such as gram flour, bajra flour, and cow dung, which are known to favor microbial proliferation (Choudhary et al., 2018; Sharma et al., 2020). In contrast, DOGROF-1, DOGROF-2, and DOGROF-4 exhibited comparatively moderate microbial populations but contained putatively identified metabolites with distinct functional relevance. DOGROF-1 showed the presence of compounds putatively identified as caffeyl alcohol, malic acid, and gibberellin-related compounds, which are associated with lignification, metabolic activity, and cell elongation (Lee et al., 2019; Kumar et al., 2021). DOGROF-2 contained putatively identified indolelactic acid and indoleacrylic acid, reported to modulate IAA pathways and promote root and shoot development (Zhao et al., 2018; Sun et al., 2023; Tang et al., 2023).

**Figure 2:**
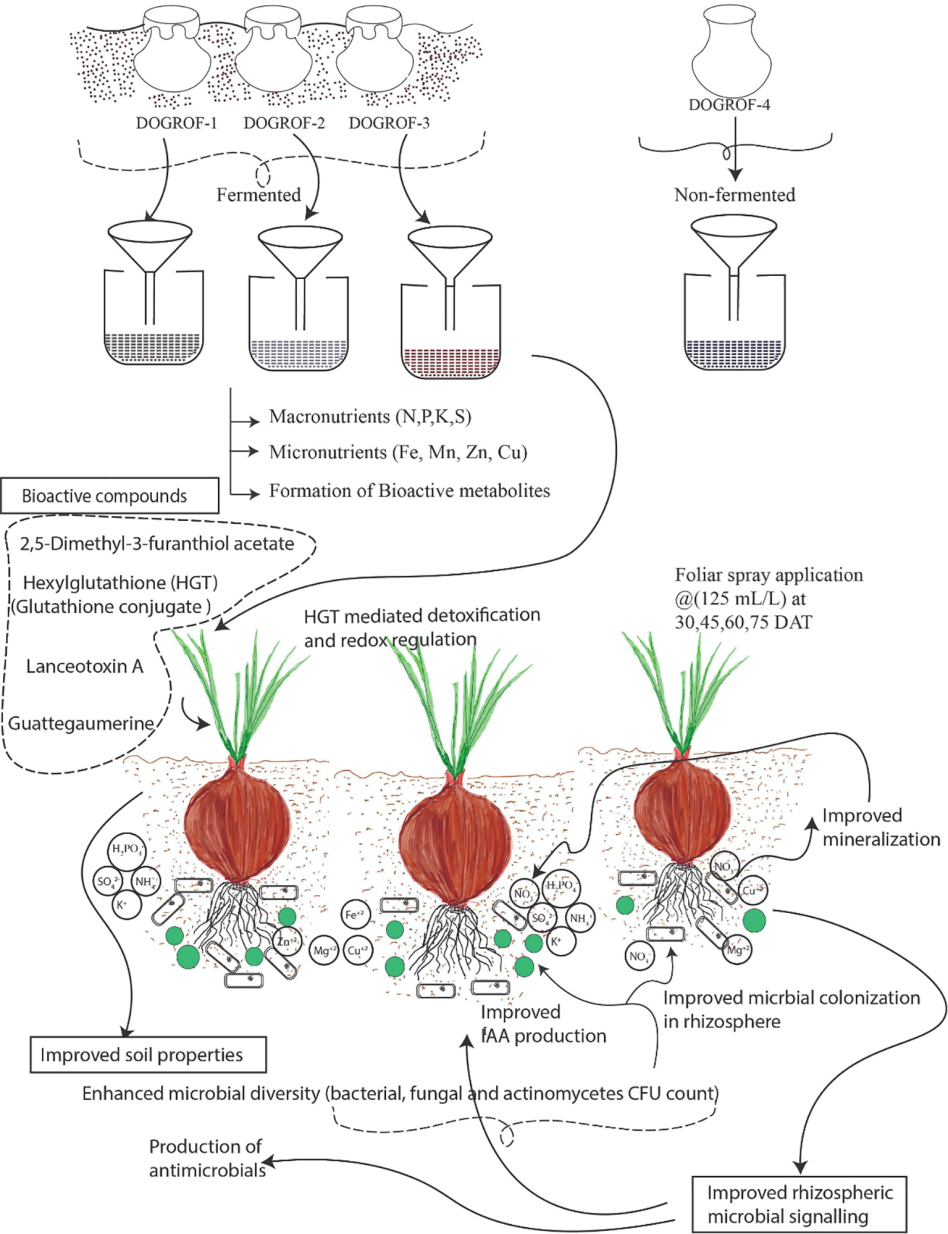
The conceptual workflow depicting the possible mode of action of bioformulations through bioactive compounds mediated oxidative stress mitigation and their effect on soil processes such as higher bacterial, fungal and actinomycete CFU count, higher mineralization (macro and micronutrients), improved microbial signaling.

DOGROF-4 exhibited tentative annotations of lupinine, isorhamnetin derivatives, and candicidin D, compounds documented for antimicrobial and antioxidant properties (Yang et al., 2017; Zhang et al., 2023; Namdar et al., 2024). Candicidin D and flavonoids such as isorhamnetin are implicated in pathogen inhibition and stress resilience (Yonekura-Sakakibara et al., 2008; Yao et al., 2021). The antifungal activity observed in DOGROF-4 may therefore be linked to these putatively identified secondary metabolites, including those potentially derived from Calotropis and neem extracts, which are known to contain bioactive compounds such as azadirachtin and uscharin (Kumar and Neeraj, 2015; Singh et al., 2019). As these annotations are based solely on LC-MS mass matching, their functional roles remain provisional. Collectively, these results highlight that microbial metabolite interactions play a pivotal role in determining the bioefficacy of organic formulations. The presence of growth regulators, redox metabolites, and antifungal compounds in conjunction with beneficial microflora may have contributed to enhanced onion crop health and yield as per the schematic proposed in **Figure 2**. Such formulations, particularly DOGROF-3, demonstrate that integrating traditional knowledge with biochemical and microbial optimization can offer cost-effective, and environmentally compatible strategies for disease management in onion cultivation (Bhattacharjya et al., 2021; Nandhini and Somasundaram, 2023). The superior performance of DOGROF-3, which resulted in 34.23% (*Kharif*) and 29.17% (*Rabi*) higher yields over control, along with disease reductions of 49.62% and 48.09%, respectively, reflects the synergistic action of its components - bajra flour, *Calotropis* and *Karanj* leaves, and spices such as turmeric and ginger. These components may improve nutrient status and could contribute to antifungal activity and enhanced plant defense mechanisms (Patel et al., 2017). Moreover, the rich microbial population and nutrient composition of DOGROF-3 may have supported root vigor, nutrient uptake, and improved physiological efficiency in onion plants (**Figure 2**).

The present findings demonstrated that foliar application of the developed organic formulations significantly improved onion growth and productivity while reducing the incidence of major foliar diseases. Among the formulations, DOGROF-3 showed superior performance in enhancing plant height, pseudostem girth, leaf number, and bulb yield during both *Kharif* and *Rabi* seasons. The improvement in vegetative growth and yield may be associated with the combined presence of diverse microbial populations and bioactive metabolites, which are known to facilitate nutrient mineralization, phytohormone production, and disease suppression. These results are in close agreement with the earlier findings of Kumar and Singh (2021), who reported that pre-harvest foliar application of organic formulations such as panchagavya and neem-based formulations (Besara) significantly increased plant height, number of leaves, bulb yield, bulb diameter, and bulb weight irrespective of the onion varieties tested under Haryana conditions. The similarity between their results and the present study supports the premise that bioenhancer formulations enriched with microbial and organic constituents stimulate plant physiological and biochemical processes, thereby improving crop performance. Bindumathi (2008) evaluated the yield and quality parameters of various organic growth promoters like panchagavya and amritpani on brinjal and tomato and observed that amritpani treated plants showed early flowering in brinjal and a larger number of flowers, followed by less flower drop in tomato when compared with panchagavya treated plants. More et al. (2008) also reported that by the application of 5 t ha-1 FYM + amritpani + PSB + rhizobium there is a significant higher value for growth attributes, yield and yield attributes of soybean when compared to other combinations such as application of farmyard manure, 5 t/ha farmyard manure + amritpani, 5 t/ha FYM + Phosphate solubilizing bacteria. Raskar and Wani (2014) observed that amritpani controls pest in paddy and is a good tonic for crop as it increases the growth of the crop. Dwivedi et al. (2014) studied the effect on vegetative parameters, flowering, and fruiting with the treatment of organic amendments like amritpani, jeevamruth + vermicompost and farmyard manure in Cape gooseberry. Sakhubai et al. (2014) studied effect of bio-inoculant mycorrhiza and organic formulations, that is panchagavya and amritpani on growth, yield and quality of buckwheat and recommended that the organic treatment (VAM + panchagavya + amritpani 3% drench and spray) for enhanced growth and yield parameters of buckwheat. In addition, several studies have demonstrated that the incorporation of Pongamia-derived bioactive compounds in organic formulations supports sustainable pest management, as karanjin has been shown to function as an efficient pesticide in organic agricultural programs where chemical inputs are restricted (Lale and Kulkarni, 2010; Degani et al., 2022). Being a leguminous plant, *Pongamia* can also form symbiotic associations with mycorrhizal fungi, further enhancing nutrient acquisition and contributing to overall soil health and crop productivity. The consistency of these results across different geographical regions and organic inputs underscores the robustness of using indigenous organic formulations as a sustainable alternative to chemical fertilizers and fungicides in onion cultivation.

The higher incremental cost benefit ratio *Kharif* and *Rabi* (1:33.65 and 1:34.04) of DOGROF-3 also underscores its economic feasibility, offering a sustainable and low-cost alternative to conventional fungicides. The results collectively emphasize that integrating traditional knowledge of organic preparation with modern analytical validation can unearth potent, eco-friendly formulations that enhance both crop productivity and resilience against diseases. Such bio-formulations could be effectively incorporated into integrated pest and disease management (IPDM) programs for onion cultivation to reduce chemical dependency and environmental risks (Singh et al., 2021). While the present findings highlight the potential functional relevance of the putatively identified metabolites and associated field efficacy of the evaluated bioformulations, important considerations related to biosafety and reproducibility needs to be considered before large scale adoption. As such bioformulations incorporate biologically active inputs such as cow dung, cow urine, plant extracts, and fermented substrates, which inherently contain complex and dynamic microbial consortia. Given the organic and fermented nature of these materials, comprehensive biosafety evaluation is essential to exclude the presence of human, animal, or plant pathogens. Studies have shown that pathogens can survive in livestock manure during storage and after field application, posing potential risks to crops and human health (Nicholson et al., 2005; Peng et al., 2024). Routine microbiological screening, pathogen-specific assays, and quality control measures would be necessary prior to large-scale application. In addition, environmental conditions during fermentation may influence microbial load and metabolite composition, potentially leading to batch-to-batch variability (Chen et al, 2025). Such batch-to-batch variability though remains concern, nonetheless the studies conducted across different locations have shown the promising results and therefore the effects are often not impacting the efficacy of bioformulations.

## 5 Conclusions

The present study revealed that organic formulations developed from locally available bio-resources significantly improved onion crop health, yield, and disease management under both *Kharif* and *Rabi* conditions. Among the four formulations, DOGROF-3 consistently exhibited superior performance, reducing anthracnose-twister and *Stemphylium* blight disease incidence with yield enhancement. Microbial enumeration indicated that DOGROF-3 and DOGROF-1 harbored higher populations of bacteria, fungi, and actinomycetes, suggesting a rich microbial consortium beneficial for nutrient transformation and disease suppression. LC-MS analysis identified several putative bioactive non-volatile organic compounds, including indoleacrylic acid, gibberellins, Guattegaumerine, Lanceotoxin A, and Ganoderic acid H and many glutathione derivatives (GST-related compounds), associated with growth promotion and antifungal activity which may underlie the potential action of such organic formulation in onion growth promotion and disease management. The high ICBR recorded for DOGROF-3 highlights its economic feasibility. The current study is an attempt to explore the field efficacy and biochemical basis of mode of action of such organic formulations, however at the same time it warrants the evaluation of such formulations for biosafety, pathogen presence and environmental implications, before their large-scale commercial production and application. These findings collectively established DOGROF-3 as an eco-friendly, and economically viable alternative to chemical inputs, offering a cost-effective approach for enhancing onion crop health and productivity under field conditions.

## Data Availability

The original contributions presented in the study are included in the article/**Supplementary Material**. Further inquiries can be directed to the corresponding authors.

## References

[B1] Ali W. M. Sultan S. M. Ali A. M. Al-Sayed H. M. Mahmoud M. A. Ismail H. G. . (2025). Organic fertilizers and Azotobacter: effects on onion growth, yield, metabolites, and soil fertility. AMB Express 15, 86. doi: 10.1186/s13568-025-01895-5. PMID: 40447833 PMC12125436

[B2] Alloway B. J. (2008). Zinc in soils and crop nutrition (Brussels: International Zinc Association).

[B3] Anandham R. Premalatha N. Jee H. J. Weon H. Y. Kwon S. W. Krishnamoorthy R. . (2015). Cultivable bacterial diversity and early plant growth promotion by the traditional organic formulations prepared using organic waste materials. Int. J. Recyl Org. Waste Agric. 4, 279–289. doi: 10.1007/s40093-015-0107-1. PMID: 41933263

[B4] Bertsch C. Ramirez-Suero M. Magnin-Robert M. Larignon P. Chong J. Abou-Mansour E. (2013). Grapevine trunk diseases: complex and still poorly understood. Plant Pathol. 62, 243–265. doi: 10.1111/j.1365-3059.2012.02674.x. PMID: 41940437

[B5] Bhardwaj G. S. Jain A. Jangid T. Jangir R. N. Sharma A. (2025). Calotropis procera: A comprehensive review of its phytochemistry, ethnomedicinal uses, and pharmacological potential. S Afr J. Bot. 185, 235–276. doi: 10.1016/j.sajb.2025.08.001. PMID: 41936479

[B6] Bhardwaj R. L. Sharma A. Meena P. Choudhary K. Kumawat S. R. Parashar A. (2025). Effect of integrated nutrient management ongrowth, yield, nutritional value, and nutraceutical quality of onion bulbs (Allium cepa L.). J. Spices Aromatic Crops 34, p81–101. doi: 10.25081/josac.2025.v34.i1.9778

[B7] Bhattacharjya S. Sahu A. Phalke D. H. Manna M. C. Thakur J. K. Mandal A. . (2021). In situ decomposition of crop residues using lignocellulolytic microbial consortia: a viable alternative to residue burning. Environ. Sci. pollut. Res. 28, 32416–32433. doi: 10.1007/s11356-021-12611-8. PMID: 33625699

[B8] Bindumathi M. (2008). Evaluation of organic growth promoters on yield of dryland vegetable crops in India. J. Organic Sys 3, 24.

[B9] Broadley M. Brown P. Cakmak I. Rengel Z. Zhao F. (2012). “ Function of nutrients: micronutrients,” in Marschner’s mineral nutrition of higher plants. Ed. Marschner P. ( Academic Press, London), 191–248.

[B10] Cakmak I. (2008). Enrichment of cereal grains with zinc: agronomic or genetic biofortification? Plant Soil 302, 1–17. doi: 10.1007/s11104-007-9466-3. PMID: 41933263

[B11] Chandel R. Kamil D. Singh S. Kumar A. Patel R. Verma P. . (2022). Screening of short-day onions for resistance to Stemphylium leaf blight in the seed-to-bulb stage (stage I) and bulb-to-seed stage (stage II). Front. Plant Sci. 13. doi: 10.3389/fpls.2022.1063685. PMID: 36466277 PMC9709266

[B12] Chejara B. K. (2013). Studies on gram pod borer, *Helicoverpa armigera* (Hub.) on chickpea and its control with insecticides and biopesticides. M.Sc. thesis. Jabalpur: Jawaharlal Nehru Krishi Vishwavidyalaya.

[B13] Chen X. Ma X. Liu Z. Gu H. Fang H. Shen Z. . (2025). Organic fertilizers increase microbial community diversity and stability slowing down the transformation process of nutrient cycling. Environ. Microbiome 20, 130. doi: 10.1186/s40793-025-00791-6. PMID: 41088478 PMC12522994

[B14] Choudhary M. Panday S. C. Meena V. S. Singh S. Yadav R. P. Mahanta D. . (2018). Long-term effects of organic manure and inorganic fertilization on sustainability and chemical soil quality indicators of soybean-wheat cropping system in the Indian mid-Himalayas. Agric. Ecosyst. Environ. 257, 38–46.

[B15] Copping L. G. Duke S. O. (2007). Natural products that have been used commercially as crop protection agents. Pest Manage. Sci. 63, 524–554. doi: 10.1002/ps.1378. PMID: 17487882

[B16] Degani E. Prasad M. V. R. Paradkar A. Pena R. Soltangheisi A. Ullah I. (2022). A critical review of *Pongamia pinnata* multiple applications: from land remediation and carbon sequestration to socioeconomic benefits. J. Environ. Manage. 324, 116297. 36174475 10.1016/j.jenvman.2022.116297

[B17] Deori M. Sadras Bhavana S. D. Meka V. A. Anup S. R. Ravi Teja N. R. Vikash K. (2025). Organic liquid formulations in horticultural crops: A comprehensive review on growth, quality, and soil health. Plant Arch. 25, 26–39. doi: 10.51470

[B18] Dhakad A. K. Kumar R. Choudhary R. Singh S. Khan S. Poonia P. K. (2025). Traditional to modern perspectives on Neem (Azadirachta indica): A gateway to bioactive compounds, sustainable agrochemicals and industrial applications. Ind. Crop Prod. 231, 121155. doi: 10.1016/j.indcrop.2025.121155. PMID: 41936479

[B19] Dutta R. K J. Nadig S. M. Manjunathagowda D. C. Gurav V. S. Singh M. (2022). Anthracnose of onion (Allium cepa L.): A twister disease. Pathogens 11, 884. doi: 10.3390/pathogens11080884. PMID: 36015005 PMC9415854

[B20] Dutta R. Kumar S. Jayalakshmi K. Radhakrishna A. Bhagat K. Manjunatha Gowda D. C. . (2024). Potential of Trichoderma strains to positively modulate plant growth processes and bulb yield in Rabi onion. Front. Sustain. Food. Syst. 8. doi: 10.3389/fsufs.2024.1427303. PMID: 41930257

[B21] Dwivedi D. H. Yadav A. K. Kumar P. Gautam S. K. (2014). Integrated nutrient management in cape gooseberry (Physalis Peruviana) for peri urban horticulture. Indian J. Appl. Res. 4, 2249–2555. doi: 10.36106/ijar. PMID: 37497239

[B22] FAO (2025). FAOSTAT statistical database (Rome: Food and Agriculture Organization of the United Nations). Available online at: https://www.fao.org/faostat/ (Accessed November 18, 2025).

[B23] Gullner G. Komives T. Király L. Schröder P. (2018). Glutathione S-transferase enzymes in plant-pathogen interactions. Front. Plant Sci. 9. doi: 10.3389/fpls.2018.01836. PMID: 30622544 PMC6308375

[B24] Hay F. S. Sharma S. Hoepting C. Strickland D. Luong K. Pethybridge S. J. (2019). Emergence of Stemphylium leaf blight of onion in New York associated with fungicide resistance. Plant Dis. 103, 3083–3092. doi: 10.1094/pdis-03-19-0676-re. PMID: 31596693

[B25] Jayaprakasha G. K. Jagan Mohan Rao L. Sakariah K. K. (2005). Chemistry and biological activities of Curcuma longa. Trends Food Sci. Technol. 16, 533–548. doi: 10.1016/j.tifs.2005.08.006. PMID: 41936479

[B26] Jwaideh M. A. Sutanudjaja E. H. Dalin C. (2022). Global impacts of nitrogen and phosphorus fertiliser use for major crops on aquatic biodiversity. Int. J. Life Cycle Assess 27, 1058–1080. doi: 10.1007/s11367-022-02078-1. PMID: 41933263

[B27] Kabaluk J. T. Svircev A. M. Goettel M. S. Woo S. G. (2010). The use and regulation of microbial pesticides in representative jurisdiction worldwide (Hong Kong, China: IOBC Global), 9.

[B28] Kabata-Pendias A. Pendias H. (2001). Trace elements in soils and plants (Boca Raton: CRC Press).

[B29] Kale R. B. Gavhane A. D. Thorat V. S. Gadge S. S. Wayal S. M. Gaikwad S. Y. . (2024). Efficiency dynamics among onion growers in Maharashtra: a comparative analysis of drip irrigation adopters and non-adopters. BMC Plant Biol. 24, 237. doi: 10.1186/s12870-024-04875-2. PMID: 38566021 PMC10988828

[B30] Kalhoro M. T. Zhang H. Kalhoro G. M. Wang F. Chen T. Faqir Y. . (2022). Fungicidal properties of ginger (Zingiber officinale) essential oils against Phytophthora colocasiae. Sci. Rep. 12, 2191. doi: 10.1038/s41598-022-06321-5. PMID: 35140298 PMC8828847

[B31] Khandare R. N. Chandra R. Pareek N. Raverkar K. P. (2020). Carrier-based and liquid bioinoculants of Azotobacter and PSB saved chemical fertilizers in wheat (Triticum aestivum L.) and enhanced soil biological properties in Mollisols. J. Plant Nutr. 43, 36–50. doi: 10.1080/01904167.2019.1659333. PMID: 41909888

[B32] Kumar S. Gupta P. K. Sharma R. K. (2021). Opportunities for research and innovation in sustainable agriculture. Sustainability 13, 5671. doi: 10.3390/agronomy15010076. PMID: 41725453

[B33] Kumar V. S. S. Neeraj S. (2015). Post-harvest management of fungal diseases in onion-a review. Int. J. Curr. Microbiol. 4737, 52. doi: 10.1201/9781003454922-3

[B34] Kumar A. D. Rahul K. Raman C. Simrat S. Salman K. Pawan K. P. (2025). Traditional to modern perspectives on Neem (Azadirachta indica): A gateway to bioactive compounds, sustainable agrochemicals and industrial applications. Ind. Crop Prod. 231, 121155. doi: 10.1016/j.indcrop.2025.121155. PMID: 41936479

[B35] Kumar C. S. Singh G. (2021). Role of Organic Liquid Formulations in Agriculture: A review. Int. J. Emerg. Technol. Innov. Res. 8, 250–255. Available online at: http://www.jetir.org/papers/JETIR2104233.pdf.

[B36] Kuzyakov Y. Blagodatskaya E. (2015). Microbial hotspots and hot moments in soil: concept and review. Soil Biol. Biochem. 83, 184–199. doi: 10.1016/j.soilbio.2015.01.025. PMID: 41936479

[B37] Lahdenpera M. L. Simon E. Uoti J. (1991). “ Mycostop-a novel biofungicide based on Streptomyces bacteria”, in: Developments in agricultural and managed forest ecology (Amsterdam, The Netherlands: Elsevier), 23, 258–263. doi: 10.1016/B978-0-444-88728-3.50048-2

[B38] Lale A. Kulkarni D. (2010). A mosquito repellent karanj Kunapa from pongamia pinnata. Asian Agric. Hist 14, 207–211.

[B39] Leclercq J. Quetin J. De Pauw-Gillet M. C. Bassleer R. Angenot L. (1987). Antimitotic and cytotoxic activities of guattegaumerine, a bisbenzylisoquinoline alkaloid. Planta Med. 53, 116–117. doi: 10.1055/s-2006-962646. PMID: 3575505

[B40] Lee M. H. Jeon H. S. Kim S. H. Chung J. H. Roppolo D. Lee H. J. . (2019). Lignin-based barrier restricts pathogens to the infection site and confers resistance in plants. EMBO J. 38 (23), EMBJ2019101948. 10.15252/embj.2019101948 PMC6885736 31559647

[B41] Li J. Li K. Li Y. Yue X. Zhu H. Shi L. . (2025). Optimizing fertilization rate to achieve high onion bulb yield and high nitrogen fertilizer productivity in dry-hot valley region of southwest China. Agronomy 15, 1822. doi: 10.3390/agronomy15081822. PMID: 41725453

[B42] Li W. J. Nie S. P. Liu X. Z. Zhang H. Yang Y. Yu Q. . (2012). Antimicrobial properties, antioxidant activity and cytotoxicity of ethanol-soluble acidic components from Ganoderma atrum. Food Chem. Toxicol. 50, 689–694. doi: 10.1016/j.fct.2011.12.011. PMID: 22198608

[B43] Lindsay W. L. (1979). Chemical equilibria in soils (New York: Wiley).

[B44] Marschner P. (2012). Marschner’s mineral nutrition of higher plants (London: Academic Press). doi: 10.1016/C2009-0-63043-9

[B45] Mauch F. Dudler R. (1993). Differential induction of distinct glutathione-S-transferases of wheat by xenobiotics and by pathogen attack. Plant Physiol. 102, 1193–1201. doi: 10.1104/pp.102.4.1193. PMID: 8278547 PMC158905

[B46] Mitra D. Mondal R. Khoshru B. Senapati A. Radha T. K. Mahakur B. . (2022). Actinobacteria-enhanced plant growth, nutrient acquisition, and crop protection: Advances in soil, plant, and microbial multifactorial interactions. Pedosphere 32, 149–170. doi: 10.1016/S1002-0160(21)60042-5. PMID: 41908327

[B47] More S. R. Mendhe S. N. Kolte H. S. Venprediwar M. D. Choudhary R. L. (2008). Growth and yield attributes of soybean as influenced by nutrient management. J. Soil Crop 18, 154–157. doi: 10.18782/2320-7051.7228

[B48] Namdar D. Mulder P. P. J. Ben-Simchon E. Hacham Y. Basheer L. Cohen O. . (2024). New analytical approach to quinolizidine alkaloids and their assumed biosynthesis pathways in lupin seeds. Toxins 16, 163. doi: 10.3390/toxins16030163. PMID: 38535829 PMC10974633

[B49] Nandhini D. U. Somasundaram E. (2023). Characterising the traditional organic liquid formulations used by the farmers of western agro-climatic zone of Tamil Nadu. Indian J. Tradit Knowl. 22, 297–306. doi: 10.56042/ijtk.v22i2.40024

[B50] Nicholson F. A. Groves S. J. Chambers B. J. (2005). Pathogen survival during livestock manure storage and following land application. Bioresour Technol. 96, 135–143. doi: 10.1016/j.biortech.2004.02.030. PMID: 15381209

[B51] Palekar S. (2006). Shoonya bandovaladanaisargikakrushi (Bangalore, India: Swamy Anand Agri Prakashana).

[B52] Patel R. D. Bharpoda T. M. Borad P. K. Bhatt N. A. Mahida R. D. (2017). Efficacy of different bio-pesticides against sucking pests of Bt cotton. AGRES An Int. e-J 6, 171–180.

[B53] Peng N. Zhang J. Hu R. Liu S. Liu F. Fan Y. . (2024). Hidden pathogen risk in mature compost: Low optimal growth temperature confers pathogen survival and activity during manure composting. J. Hazard Mater. 480, 136230. doi: 10.1016/j.jhazmat.2024.136230. PMID: 39442307

[B54] Prasad S. Aggarwal B. B. (2011). “ Turmeric, the golden spice: from traditional medicine to modern medicine,” in Herbal medicine: biomolecular and clinical aspects. Eds. Benzie I. F. F. Wachtel-Galor S. (Boca Raton (FL): CRC Press). 22593922

[B55] Qiu D. Wang Y. Xu N. Chen B. Zhu Y. Zhang Z. . (2025). Global variation in plant-beneficial bacteria in soil under pesticide stress. Nat. Commun. 16, 10685. doi: 10.1038/s41467-025-65719-7. PMID: 41309644 PMC12661013

[B56] Ram R. A. (2023). On-farm organic inputs generation for quality vegetable production. In: Vegetables for nutrition and entrepreneurship. 115–140. Singapore: Springer Nature Singapore.

[B57] Raskar S. S. Wani A. G. (2014). Promotion of organic farming in tribal farmers of akole with relation to climate change. Int. J. Curr. Res. 6, 4697–4701.

[B58] Sakhubai H. T. Laxminarayana H. Chaya P. (2014). Effect of bio-inoculants and bioformulations on growth, yield and quality of buckwheat. Int. J. Agric. Sci. Vet. Med. 2, 2320–3730.

[B59] Sharma S. K. Jain D. Choudharya R. Jata G. Jain P. Bhojiya A. A. . (2021). Microbiological and enzymatic properties of diverse jaivik krishi inputs used in organic farming. Indian J. Tradit Knowl. 20, 237–243. doi: 10.4018/978-1-5225-6111-8.ch022

[B60] Sharma I. P. Kanta C. Dwivedi T. Rani R. (2020). “ Indigenous agricultural practices: A supreme key to maintaining biodiversity,” in Microbiological advancements for higher altitude agro-ecosystems & Sustainability. Rhizosphere biology. Eds. Goel R. Soni R. Suyal D. ( Springer, Singapore). doi: 10.1007/978-981-15-1902-4_6

[B61] Singh G. Kapoor I. P. S. Pandey S. K. Singh U. K. Singh R. K. (2002). Studies on essential oils: part 10 - antibacterial activity of volatile oils of some spices. Phytotherapy Res. 16, 680–682. doi: 10.1002/ptr.951. PMID: 12410554

[B62] Singh V. Keswani C. Ray S. Upadhyay R. S. Dhananjaya P. Singh P. R. (2019). Isolation and screening of high salinity tolerant Trichoderma spp. with plant growth property and antagonistic activity against various soilborne phytopathogens. Arch. Phytopathol. Plant Protec 52, 667–680. doi: 10.1080/03235408.2019.1648917. PMID: 41909888

[B63] Singh S. Kumar V. Dhanjal D. S. Dhaka S. V. Thotapalli S. Singh J. . (2021). “ Rhizosphere biology: A key to agricultural sustainability,” in Current Trends in Microbial Biotechnology for Sustainable Agriculture. Environmental and microbial biotechnology. Eds. Yadav A. N. Singh J. Singh C. Yadav N. ( Springer Nature, Singapore). doi: 10.1007/978-981-15-6949-4_7

[B64] Sodani R. Kumar S. (2017). Cow urine: a boon for sustainable agriculture. Int. J. Curr. Microbiol. App Sci. 6, 1824–1829. doi: 10.20546/ijcmas.2017.602.205

[B65] Subbarao N. S. (1999). Soil microorganisms and plant growth (New Delhi, India: Oxford and IBH publishing Company), 1–333.

[B66] Sun P. Huang Y. Yang X. Liao A. Wu J. (2023). The role of indole derivative in the growth of plants: A review. Front. Plant Sci. 13. doi: 10.3389/fpls.2022.1120613. PMID: 36726683 PMC9885212

[B67] Tang J. Li Y. Zhang L. Mu J. Jiang Y. Fu H. . (2023). Biosynthetic pathways and functions of indole-3-acetic acid in microorganisms. Microorganisms 11, 2077. doi: 10.3390/microorganisms11082077. PMID: 37630637 PMC10459833

[B68] Torres-Rodriguez J. A. Reyes-Pérez J. J. Quiñones-Aguilar E. E. Hernandez-Montiel L. G. (2022). Actinomycete potential as biocontrol agent of phytopathogenic fungi: mechanisms, source, and applications. Plants 11, 3201. doi: 10.3390/plants11233201. PMID: 36501241 PMC9736024

[B69] Usha K. Singh B. Praseetha P. Deepa N. Agarwal D. K. Agarwal R. . (2009). Antifungal activity of Datura stramonium, Calotropis gigantea and Azadirachta indica against Fusarium mangiferae and floral malformation in mango. Eur. J. Plant Pathol. 124, 637–657. doi: 10.1007/s10658-009-9450-2. PMID: 41933263

[B70] Wheeler B. E. J. (1969). An introduction to plant diseases. London: Wiley. 374.

[B71] Yagmur A. Demir S. Canpolat S. Rezaee Danesh Y. Farda B. Djebaili R. . (2024). Onion Fusarium basal rot disease control by arbuscular mycorrhizal fungi and Trichoderma harzianum. Plants 13, 386. doi: 10.3390/plants13030386 38337919 PMC10857072

[B72] Yang S. S. Cheng M. J. Chan H. Y. Hsieh S. Y. Wu H. C. Yuan G. F. (2017). New secondary metabolites from an endophytic fungus in Porodaedalea pini. Rec Nat. Prod. 11, 251–257. doi: 10.1007/s10600-024-04501-5. PMID: 41933263

[B73] Yao W. Liu K. Liu H. Jiang Y. Wang R. Wang W. (2021). A valuable product of microbial cell factories: microbial lipase. Front. Microbiol. 12. doi: 10.3389/fmicb.2021.743377. PMID: 34616387 PMC8489457

[B74] Yemata G. Mengistu E. (2024). Potential of cattle urine as an alternative fertilizer for maize (Zea mays L.) production in Ethiopia. Heliyon 10, e39111. doi: 10.1016/j.heliyon.2024.e39111. PMID: 39619593 PMC11605349

[B75] Yonekura-Sakakibara K. Tohge T. Matsuda F. Nakabayashi R. Takayama H. Niida R. . (2008). Comprehensive flavonol profiling and transcriptome coexpression analysis leading to decoding gene–metabolite correlations in Arabidopsis. Plant Cell 20, 2160–2176. doi: 10.1105/tpc.108.058040. PMID: 18757557 PMC2553606

[B76] Yuvasri E. A. Anandham R. Balachandar D. Senthilkumar M. Thiyageshwari S. Vincent S. (2024). Harnessing the power of traditional organic formulations for crop growth and microbial harmony. Front. Bioscience-Elite 16, 14. doi: 10.31083/j.fbe1602014. PMID: 38939912

[B77] Zhang T. Huanhuan L. Ting Y. Sabry M. S. Yingqi N. Shiyu X. . (2023). Organic matter stabilization and phosphorus activation during vegetable waste composting: Multivariate and multiscale investigation. Sci. Total Environ. 891, 164608. doi: 10.1016/j.scitotenv.2023.164608. PMID: 37286002

[B78] Zhang S. Wei J. Zhang J. Chen M. Zhang Y. Cai Y. . (2025). Combined applications of organic bran Fertilizer, biochar and microbial inoculants control tobacco soil-borne diseases by recruiting beneficial rhizosphere microbes and enhancing soil quality. Biol. Control 212, 105948. doi: 10.1016/j.biocontrol.2025.105948. PMID: 41936479

[B79] Zhao F. Z. Ren C. J. Han X. H. Yang G. H. Wang J. Doughty R. (2018). Changes of soil microbial and enzyme activities are linked to soil C, N and P stoichiometry in afforested ecosystems. Forest Ecol. Manage. 427, 289–295. doi: 10.1016/j.foreco.2018.06.011. PMID: 41936479

