# Supplementary material for: Organic formulation modulating crop health, yield and managing anthracnose-twister and Stemphylium blight of onion
Source: Front Plant Sci. 2026 May 11;17:1774060. doi: 10.3389/fpls.2026.1774060 (PMC13199324; doi:10.3389/fpls.2026.1774060)
Supplement: Supplementary file 1 [file DataSheet1.docx]

**Table S1: Disease rating scale for *Stemphylium* assessment**

| **Rating scale** | **Description** | **Corresponding % damage** |
| --- | --- | --- |
| 0 | No disease | No disease |
| 1 | A few light yellow to tan, water-soaked lesions covering less than 10% leaf area | 1-10 |
| 2 | Several dark brown or black spots covering less than 20% leaf area | 11-20 |
| 3 | Dark olive-brown to black spots, covering up to 30% leaf area | 21-30 |
| 4 | Long streaks covering up to 50% of leaf area or breaking of leaves/stalks from the centre | 31-50 |
| 5 | Complete drying of the leaves/ stalks or breaking of the leaves/stalks from the base. | 51-100 |

**Table S2. Disease rating scale for anthracnose development assessment.**

| **Rating scale** | **Description** | **Corresponding % damage** |
| --- | --- | --- |
| 1 | Small white specks | 0.1-1 |
| 2 | Chlorotic spots | 1.1-2 |
| 3 | Advancement of the lesion concentric rings | 2.1-6 |
| 4 | A mature lesion with salmon/orange colored conidial mass | 3.1-11 |
| 5 | Lesions began to coalesce | 11.1-21 |
| 6 | Advanced lesion leading to death leaf blades | 21.1-31 |
| 7 | Most advance stage lesions, the dieback appearance of the plant leaving few leaf blades unaffected | 31.1-41 |
| 8 | Advanced lesions on the neck region | 41.1-61 |
| 9 | Complete infection, the death of the plant / Black fruiting bodies on the entire bulb | 61.1-100 |

**Table S3. Effect of organic formulations (DOGROF-1 to DOGROF-4) on onion crop health during *Kharif* seasons of 2021-2023**

| **Treatments** | **Plant height (cm)** | | | | **No. of leaves/plant** | | | | **Pseudo stem girth (mm)** | | | |
| --- | --- | --- | --- | --- | --- | --- | --- | --- | --- | --- | --- | --- |
|  | **2021** | **2022** | **2023** | **Pooled** | **2021** | **2022** | **2023** | **Pooled** | **2021** | **2022** | **2023** | **Pooled** |
| DOGROF-1 | 40.73^b^ ±2.53 | 44.40^cd^ ±1.22 | 39.02 ±3.25 | 41.38^bc^  ±1.72 | 6.53^ab^ ±0.81 | 6.47  ±0.23 | 5.00^cd^ ±0.00 | 6.00 ^bc^ ±0.20 | 11.82±1.60 | 10.84^abc^ ±0.24 | 11.31  ±2.23 | 11.32^c^ ±0.30 |
| DOGROF-2 | 42.20^b^ ±3.99 | 48.80^abc^ ±3.65 | 38.43 ±3.24 | 43.15^b^ ±4.23 | 6.42^ab^ ±0.91 | 6.33  ±0.50 | 5.38^bcd^ ±1.08 | 6.04 ^bc^ ±0.25 | 11.75±1.56 | 11.45^abc^ ±0.46 | 10.35  ±0.30 | 11.18^c^ ±0.69 |
| DOGROF-3 | 49.12^a^ ±2.57 | 53.33^a^  ±2.89 | 43.11 ±3.68 | 48.52^a^ ±2.17 | 6.67^a^ ±0.76 | 7.00  ±0.00 | 7.00^a^ ±0.00 | 6.89 ^a^ ±0.25 | 14.07  ±0.37 | 12.88^a^ ±0.57 | 12.31  ±0.27 | 13.09^a^ ±0.27 |
| DOGROF-4 | 44.33^b^±1.85 | 49.33^ab^ ±0.58 | 41.62 ±1.37 | 45.09 ^ab^ ±1.18 | 6.41^ab^ ±0.35 | 6.47  ±1.10 | 6.67^ab^ ±0.58 | 6.51^ab^ ±0.21 | 13.23±1.87 | 12.33^ab^ ±0.58 | 11.13  ±0.19 | 12.23^b^ ±0.52 |
| Existing practice | 42.21^b^±1.09 | 45.33^bcd^ ±2.52 | 40.0 0 ±0.0 | 42.52^bc^  ±1.20 | 6.27^ab^ ±0.99 | 6.42  ±0.68 | 6.17^abc^ ±0.29 | 6.29^ab^ ±0.07 | 11.45±2.33 | 11.38^abc^ ±2.59 | 11.49  ±1.83 | 11.44^bc^ ±0.20 |
| Farmers Practice | 42.15^b^±1.87 | 44.77^bcd^ ±2.64 | 42.66 ±5.11 | 43.19^b^ ±2.37 | 5.20^bc^ ±0.35 | 6.00  ±1.14 | 5.33 ^bcd^ ±0.58 | 5.51^cd^ ±0.38 | 11.12±1.29 | 10.49^bc^ ±0.50 | 10.23  ±0.40 | 10.61^c^ ±0.72 |
| Untreated control | 36.14^c^±1.99 | 42.40^d^ ±2.88 | 36.72 ±3.01 | 38.42^a^ ±1.08 | 4.80^c^ ±0.80 | 5.93  ±1.50 | 4.00 ^d^ ±0.87 | 4.91^d^ ±0.82 | 10.99±0.44 | 9.40^c^ ±0.15 | 7.86  ±1.32 | 9.41^d^  ±0.37 |

**Table S4. Effect of organic formulations (DOGROF-1 to DOGROF-4) on onion crop health during *Rabi* seasons of 2021-2023**

| **Treatments** | **Plant height (cm)** | | | | | **No. of leaves/plant** | | | | | **Pseudo stem girth (mm)** | | | | |
| --- | --- | --- | --- | --- | --- | --- | --- | --- | --- | --- | --- | --- | --- | --- | --- |
|  | **2021** | **2022** | **2023** | **Pooled** | **2021** | | **2022** | **2023** | **Pooled** | **2021** | | **2022** | **2023** | **Pooled** |  |
| DOGROF-1 | 53.30^b^ ±0.76 | 49.07^abc^±4.70 | 50.53^a^ ±3.44 | 47.52^ab^ ±1.05 | 9.13^ab^ ±0.56 | | 7.93^ab^ ±0.29 | 6.87^ab^ ±0.24 | 7.98 ^bc^ ±0.22 | 15.85^a^ ±0.33 | | 13.76^a^ ±0.27 | 13.90^ab^ ±0.70 | 14.51^a^ ±0.23 |  |
| DOGROF-2 | 54.24^ab^ ±1.71 | 49.93^ab^±3.77 | 48.13^a^ ±2.02 | 47.32^ab^ ±1.86 | 9.40^ab^ ±0.20 | | 7.53^ab^ ±0.17 | 6.67^ab^ ±0.26 | 7.87 ^bc^ ±0.17 | 15.90^a^ ±0.53 | | 13.69^a^ ±0.96 | 14.18 ^ab^ ±0.04 | 14.59^a^ ±0.19 |  |
| DOGROF-3 | 56.81^a^ ±1.29 | 52.73 ^a^ ±2.41 | 50.67^a^ ±1.36 | 48.47^a^ ±0.66 | 10.00^a^ ±0.23 | | 8.13^a^ ±0.17 | 7.80^a^ ±0.75 | 8.64 ^a^ ±0.12 | 16.21^a^ ±0.21 | | 14.37^a^ ±0.21 | 14.41^a^ ±0.83 | 15.00^a^ ±0.27 |  |
| DOGROF-4 | 55.35^ab^ ±2.34 | 49.53^abc^±4.30 | 51.13^a^ ±1.10 | 48.78^a^ ±0.49 | 9.73^ab^ ±0.17 | | 7.60^ab^ ±0.20 | 7.27^a^ ±0.26 | 8.20 ^ab^ ±0.10 | 16.04^a^ ±0.12 | | 13.79^a^ ±0.59 | 14.15^ab^ ±1.05 | 14.66^a^ ±0.40 |  |
| Existing practice | 46.79^c^ ±2.91 | 45.13^bcd^±1.92 | 42.00^b^±2.64 | 45.42^b^ ±0.53 | 8.87^bc^ ±0.46 | | 7.81^ab^ ±0.33 | 7.33^a^ ±0.33 | 8.00 ^bc^ ±0.04 | 15.65^a^ ±0.24 | | 12.91^ab^ ±0.48 | 13.11^ab^ ±1.30 | 13.89 ^ab^ ±0.67 |  |
| Farmers Practice | 43.95^c^ ±0.51 | 43.71^cd^±2.52 | 41.47^b^±2.50 | 44.91^b^ ±0.31 | 8.00^c^ ±0.00 | | 7.49^ab^ ±0.49 | 7.20^a^ ±0.20 | 7.56 ^c^ ±0.23 | 14.56^b^ ±0.29 | | 11.83^bc^ ±0.44 | 12.51^ab^ ±0.25 | 12.97^b^ ±0.31 |  |
| Untreated control | 37.35^d^ ±0.99 | 41.17^d^±01.12 | 38.75^c^ ±0.43 | 38.00^c^ ±0.41 | 5.80^d^ ±0.11 | | 7.00^b^ ±0.00 | 6.00^c^ ±0.00 | 6.27 ^d^ ±0.03 | 9.89^c^ ±0.17 | | 11.17^c^ ±0.16 | 11.61^b^ ±0.30 | 10.89^c^ ±0.09 |  |

**Table S5. Effect of organic formulations (DOGROF-1 to DOGROF-4) on anthracnose-twister disease and bulb yield during *kharif* seasons of 2021-2023**

| **Treatments** | **Anthracnose-twister disease severity (%)** | | | | **PDC** | **Yield (t/ha)** | | | |
| --- | --- | --- | --- | --- | --- | --- | --- | --- | --- |
|  | **2021** | **2022** | **2023** | **Pooled** |  | **2021** | **2022** | **2023** | **Pooled** |
| DOGROF-1 | 26.00^b^ ±12.16 | 45.19^b^ ±5.13 | 43.70^a^ ±3.90 | 38.30^b^ ±6.93 | 38.77 | 16.25 ^ab^ ±3.65 | 16.07±4.18 | 16.2 ^ab^ ±1.69 | 16.19 ^abc^ ±1.81 |
| DOGROF-2 | 28.00^b^ ±3.46 | 48.89^c^ ±5.45 | 46.30^b^ ±6.11 | 41.06^bc^ ±7.62 | 30.79 | 17.29 ^a^ ±1.72 | 14.08±1.35 | 16.38 ^ab^±0.12 | 15.92 ^abc^ ±0.85 |
| DOGROF-3 | 12.67^a^ ±5.03 | 40.37^a^ ±13.44 | 41.48^a^±8.91 | 31.51^a^±4.42 | 49.62 | 20.21 ^ab^ ±0.47 | 16.38±2.50 | 17.89 ^a^ ±2.60 | 18.16 ^a^ ±1.08 |
| DOGROF-4 | 26.67^b^ ±6.11 | 45.56^bc^ ±9.69 | 42.22^a^ ±7.69 | 38.15^b^ ±7.19 | 39.01 | 18.57 ^ab^ ±0.47 | 14.17±0.62 | 16.99 ^ab^ ±0.21 | 16.57 ^ab^ ±0.51 |
| Existing practice | 28.67^b^  ±10.26 | 53.33^c^ ±2.52 | 46.63^b^ ±2.96 | 42.88^bc^ ±4.16 | 31.45 | 16.90 ^ab^ ±5.38 | 14.08±.262 | 15.38 ^b^ ±1.20 | 15.46 ^abc^ ±2.52 |
| Farmers Practice | 36.00^c^  ±11.13 | 59.26^c^ ±8.34 | 47.33^ab^ ±1.55 | 47.53^c^ ±6.26 | 24.00 | 15.07 ^ab^ ±1.87 | 13.87±3.24 | 15.29 ^b^ ±0.10 | 14.74 ^bc^ ±1.73 |
| Untreated control | 61.33^d^  ± 19.0 | 70.37^d^ ±7.56 | 55.93^c^ ±3.90 | 62.54^d^±2.71 | 0.00 | 13.11 ^b^ ±3.03 | 12.78±2.08 | 14.69 ^b^ ±0.67 | 13.53 ^c^ ±0.42 |

**Table S6. Effect of organic formulations (DOGROF-1 to DOGROF-4) on *Stemphylium* leaf blight disease and bulb yield during *rabi* seasons of 2021-2023**

| **Treatments** | ***Stemphylium* leaf blight (%)** | | | | **Yield (t/ha)** | | | |
| --- | --- | --- | --- | --- | --- | --- | --- | --- |
|  | **2021** | **2022** | **Pooled** | **PDC** | **2021** | **2022** | **2023** | **Pooled** |
| DOGROF-1 | 54.00^c^ ±3.05 | 34.67 ^a^ ± 3.33 | 44.33^c^ ±3.17 | 27.32 | 20.39 ^cd^ ±0.29 | 26.13 ^a^ ±0.71 | 23.02a±1.50 | 23.18 ^b^ ±0.29 |
| DOGROF-2 | 60.67^c^ ±0.67 | 35.33^a^ ±2.90 | 48.00^b^ ±1.52 | 21.31 | 23.18 ^bc^ ±0.18 | 27.51 ^a^ ±1.82 | 22.77a±1.14 | 24.49 ^b^ ±1.01 |
| DOGROF-3 | 34.00^a^ ±1.15 | 29.33^a^ ±1.33 | 31.67^a^ ±1.20 | 48.09 | 29.76 ^a^ ±0.77 | 27.87 ^a^ ±1.10 | 25.48^a^±1.79 | 27.70 ^a^ ±1.20 |
| DOGROF-4 | 40.00^ab^ ±1.15 | 32.67^a^ ±5.45 | 36.33^a^ ±3.28 | 40.44 | 24.61 ^b^ ±1.81 | 27.54 ±2.91 | 19.93 ^b^ ±2.72 | 24.03 ^b^ ±0.93 |
| Existing practice | 43.11^b^ ±4.49 | 45.83^b^ ±1.0 | 44.47^c^ ±2.62 | 27.09 | 23.73 ^bc^ ±0.88 | 26.07 ^a^ ±0.79 | 23.16 ^a^ ±3.24 | 24.32 ^b^ ±0.79 |
| Farmers Practice | 43.78^b^ ±2.91 | 46.17^b^ ±1.17 | 44.97^c^±2.02 | 26.28 | 21.51 ^bcd^ ±1.18 | 26.04 ^a^ ±0.49 | 21.20^a^±1.19 | 22.91 ^b^ ±0.78 |
| Untreated control | 74.00^d^ ±3.05 | 48.00^b^ ±1.15 | 61.00 ^d^ ±2.0 | 0.00 | 18.55 ^d^ ±1.63 | 25.95 ^a^ ±0.78 | 19.84^b^±1.24 | 21.44 ^b^ ±1.05 |


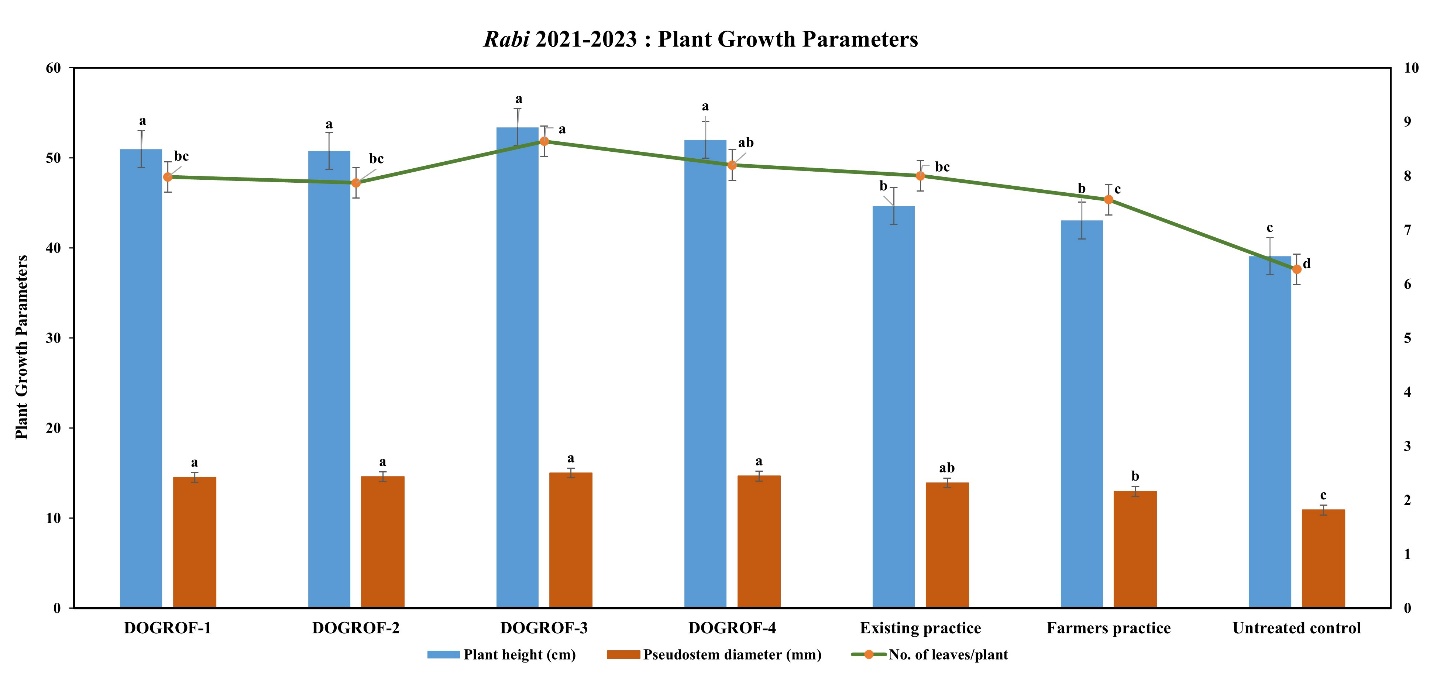


**Figure S1: Effect of application of organic formulations on the crop health of onion (pooled mean of *Rabi* 2021-2023)**


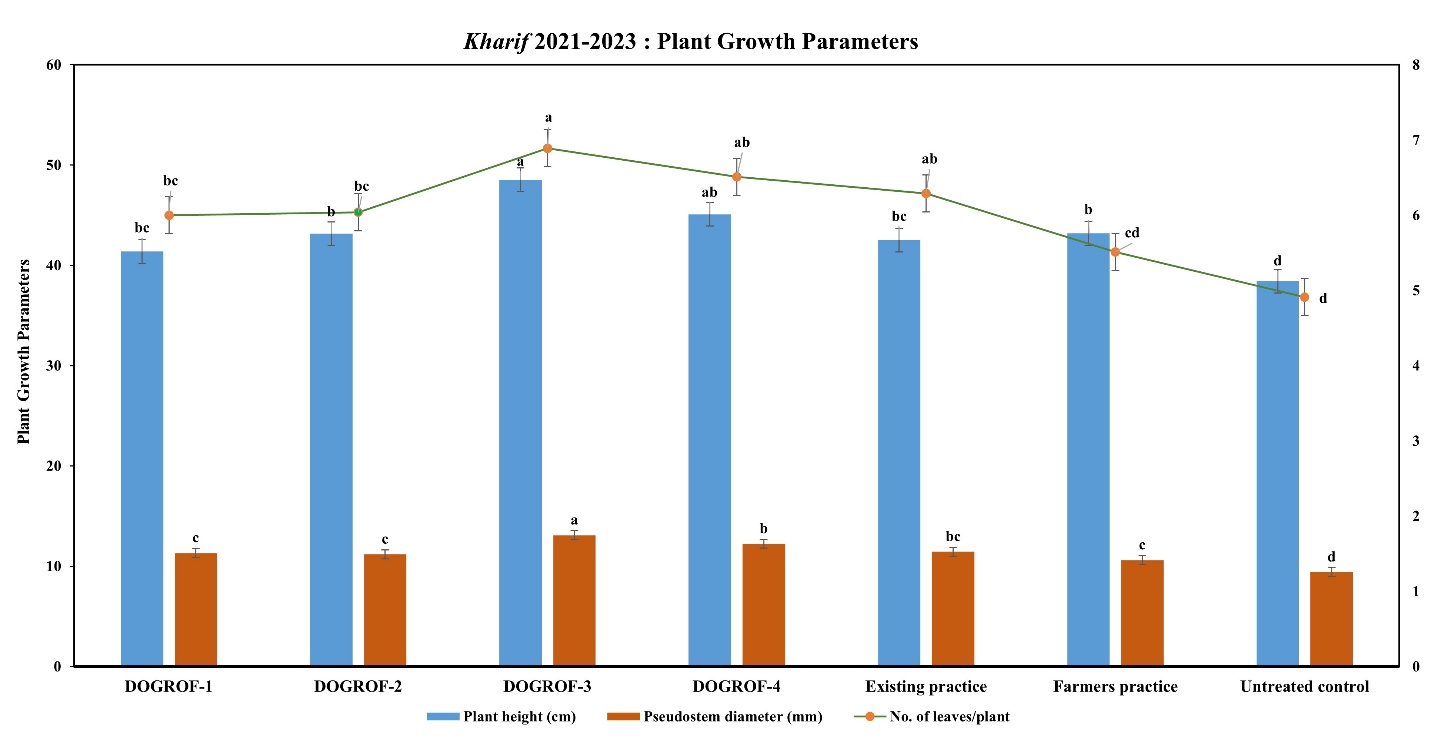


**Figure S2: Effect of application of organic formulations on the crop health of onion (pooled mean of *Kharif* 2021-2023)**


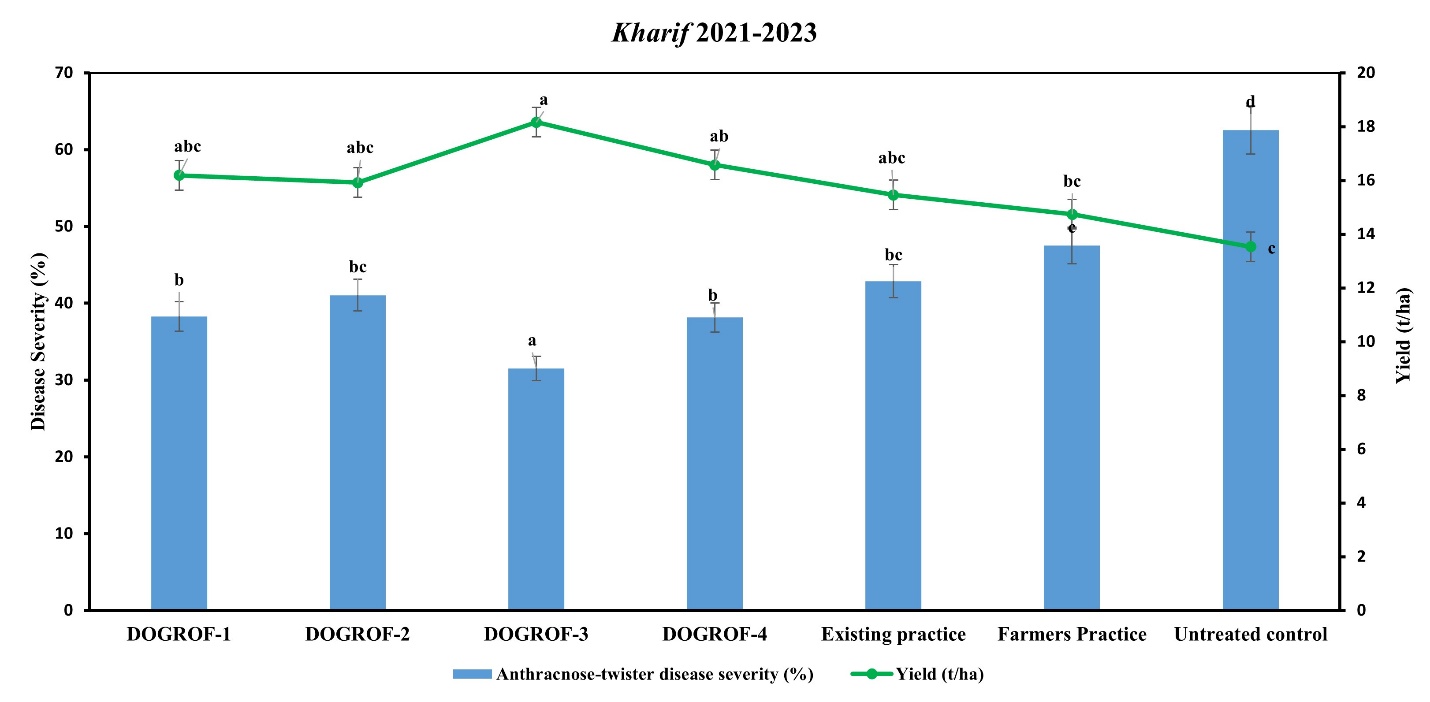


**Figure S3: Effect of application of organic formulations on Anthracnose-twister disease severity on onion (pooled mean of *Kharif* 2021-2023)**


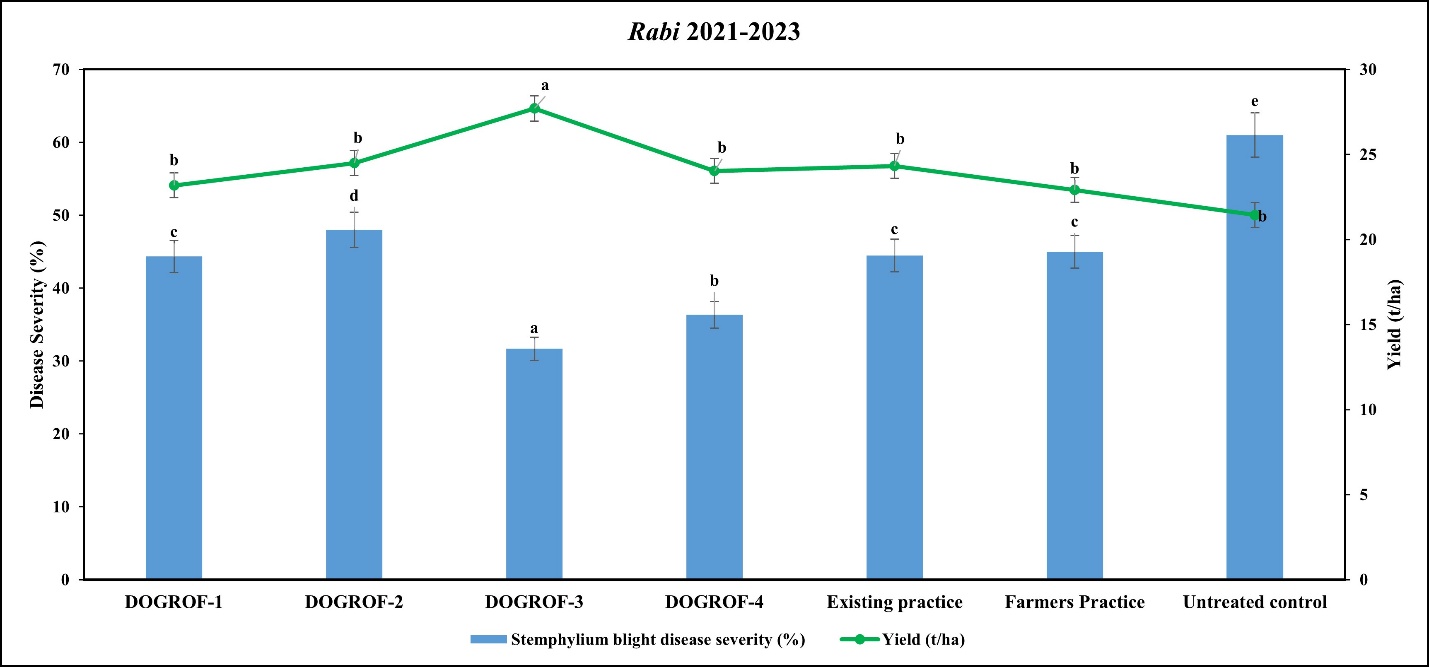


**Figure S4: Effect of application of organic formulations on *Stemphylium* blight disease severity on onion (pooled mean of *Rabi* 2021-2023)**
